# PDPN^+^ CAFs facilitate the motility of OSCC cells by inhibiting ferroptosis via transferring exosomal lncRNA FTX

**DOI:** 10.1038/s41419-023-06280-3

**Published:** 2023-11-22

**Authors:** Yaoyin Li, Zeyi Ma, Weiyu Li, Xiaoqing Xu, Peiqi Shen, Si-en Zhang, Bin Cheng, Juan Xia

**Affiliations:** 1 Hospital of Stomatology, Guanghua School of Stomatology, Sun Yat-sen University, Guangzhou, Guangdong PR China; 2 Guangdong Provincial Key Laboratory of Stomatology, Guangzhou, Guangdong PR China

**Keywords:** Cancer microenvironment, Oral cancer

## Abstract

Cancer-associated fibroblasts (CAFs) are abundant and heterogeneous in tumor microenvironment (TME). Cross-talk between cancer cells and CAFs results in cancer progression. Here, we demonstrated that a distinct cancer-associated fibroblasts subset with podoplanin (PDPN) positive expression (PDPN^+^ CAFs) was correlated with poor survival in oral squamous cell carcinoma (OSCC). PDPN^+^ CAFs promoted the progression of OSCC by transferring exosomal lncRNA FTX to OSCC cells. Mechanically, FTX bound to flap endonuclease-1 (FEN1), forming an RNA‒protein complex. FTX enhanced promoter demethylation of FEN1 by recruiting ten-eleven translocation-2 (TET2). In addition, FTX/FEN1 axis promoted OSCC cells motility by inhibiting ferroptosis. In xenograft experiments, RSL-3, a ferroptosis-inducing agent, suppressed the tumorigenesis potential of FEN1-overexpressed OSCC cells. Furthermore, Acyl-CoA synthetase long-chain family member 4 (ACSL4) was confirmed to participate in the motility promotion induced by FEN1 overexpression. FEN1 could bind to promoter region of ACSL4 and then inhibit ferroptosis in OSCC cells. Our study reveals that PDPN^+^ CAFs promote the invasiveness of OSCC cells by inhibiting ferroptosis through FTX/FEN1/ACSL4 signaling cascade. PDPN^+^ CAFs may serve as a novel potential therapeutic target for OSCC.

## Introduction

Carcinoma-associated fibroblasts (CAFs) are activated fibroblasts that exhibit distinct tumorigenic properties in many types of malignancies [1]. CAFs are correlated with tumor aggressiveness and therapy resistance, which suggest that their role is multifactorial. In oral squamous cell carcinoma (OSCC), the presence of high numbers of CAFs is associated with lymphatic invasion and poor overall survival; however, the underlying mechanism remains unclear [2]. CAFs are heterogeneous, as populations contain different subpopulations with distinct phenotypes and functions, and they promote many of the “hallmarks of malignancy” [2]. Therefore, it is necessary to identify distinct CAF subsets to understand the mechanisms by which CAFs affect OSCC progression.

Exosomes are secreted by many cell types and are extracellular vesicles (EVs) with a typical diameter of 30–150 nm [3]. In the tumor microenvironment (TME), exosomes contain proteins, lipids, mRNAs, DNA, and noncoding RNAs (ncRNAs) and mediate the horizontal transfer of information to specific recipient cells via diffusion into neighboring cells or distant anatomical organs through systemic transport [4]. The involvement of exosomal ncRNAs in tumor progression has been increasingly documented. Our previous study revealed that exosome-miR-34a-5p derived from CAFs is involved in the tumorigenesis of OSCC cells via AKT/GSK-3β/β-catenin signaling pathway [5]. Papillary thyroid carcinoma stem-like cell (PTC-CSC)-derived exosomal lncRNA DOCK9-AS2 activates Wnt/β-catenin pathway to promote PTC progression [6]. Moreover, many reports have demonstrated that exosomal lncRNAs transmit signals and phenotypes between cancer cells and CAFs [7, 8].

Long noncoding RNAs (lncRNAs) contain over 200 nucleotides and have low potential for protein coding. Many studies have shown that lncRNAs are involved in embryonic development and tumor development. LncRNA CCDST inhibits cell aggressiveness and angiogenesis in HPV-positive cervical cancer cell lines by disrupting the interaction between MDM2 and DHX9 [9]. LncRNA HOXA11-AS is upregulated in OSCC and promotes the invasiveness of OSCC cells via HOXA11-AS/miR-98-5p/YBX2 axis [10]. Ferroptosis is a newly discovered form of nonapoptotic cell death that is triggered via iron-dependent lipid peroxidation [11]. This form of cell death is characterized by increased levels of intracellular reactive oxygen species (ROS) and iron dependency, and lacks the typical manifestations of apoptosis and necrosis [12]. Therefore, ferroptosis induction may be an effective antitumor strategy for OSCC. It is well established that lncRNAs inhibit ferroptosis in different cancers. LINC00336 inhibits ferroptosis in lung cancer through interacting with the RNA-binding protein ELAVL1 [13]. LncRNA OIP5-AS1 promotes prostate cancer progression and ferroptosis resistance through miR-128-3p/SLC7A11 signaling [14]. However, the mechanism of ferroptosis-related lncRNAs in regulating OSCC progression has not been clarified, and their significance in OSCC treatment needs to be further elucidated.

Human podoplanin (PDPN) is a type 1 transmembrane sialomucin-like glycoprotein that is upregulated in several cancer types. In lung adenocarcinoma, podoplanin expression in CAFs predicts poor prognosis [15], and podoplanin-expressing CAFs promote cancer cell invasiveness via RhoA activation in lung adenocarcinoma [16]. In the current study, we demonstrated that podoplanin-positive cancer-associated fibroblasts (PDPN^+^ CAFs) were correlated with poor prognosis in OSCC. PDPN^+^ CAFs facilitated the aggressiveness of OSCC cells via exosomal lncRNA FTX, and inhibitted ferroptosis of OSCC cells through FTX/FEN1/ACSL4 signaling cascade. Our present study suggests that PDPN^+^ CAFs may serve as a novel potential therapeutic target in OSCC.

## Materials and methods

### Patients and isolation of primary human fibroblasts

Fresh OSCC (*n* = 32) and paraffin-embedded OSCC samples (*n* = 40) were obtained from the Department of Craniofacial Surgery, Guanghua School of Stomatology, Sun Yat-sen University (Guangzhou, China) between 2012 and 2021. Isolation and culture of primary human fibroblasts were performed as previously described [5]. Primary fibroblasts isolated from tumor tissues were termed cancer-associated fibroblasts (CAFs), while those from adjacent normal tissues were termed normal fibroblasts (NFs). Flow cytometry was used to isolate podoplanin positive CAFs as described previously [17]. The use of these clinical samples was approved by the Ethics Committee of Guanghua School of Stomatology, Hospital of Stomatology, Sun Yat-sen University (Approval number: KQERC-2022-92-02).

### Cell lines and culture conditions

Human oral keratinocytes (HOKs) were purchased from ScienCell and cultured in oral keratinocyte medium according to the manufacturer’s instructions. OSCC cells, including WSU-HN6, CAL27, CAL33, UM1, SAS, and SCC15, were kindly donated by Guangdong Provincial Key Laboratory of Stomatology (Guangzhou, China). HEK 293T cell was purchased from the National Centre for Cell Science (NCCS). WSU-HN6, CAL27, CAL33, HSC3, and HEK 293T cells were incubated in Dulbecco’s modified Eagle’s medium (DMEM; Gibco) supplemented with 10% FBS (fetal bovine serum; Gibco), while UM1, SCC15, and SAS cells were maintained in DMEM/F12 (Gibco) supplemented with 10% FBS (Gibco). All cells were incubated at 37 °C and 5% CO_2_.

### Preparation of lentiviral vector and transfection

The lncRNA FTX overexpression plasmid (FTX-OE), shRNA targeting lncRNA FTX (sh-FTX), PDPN overexpression plasmid (PDPN-OE), shRNA targeting PDPN (sh-PDPN), FEN1 overexpression plasmid (FEN1-OE), shRNA targeting FEN1 (sh-FEN1), ACSL4 overexpression plasmid (ACSL4-OE), shRNA targeting ACSL4 (sh-ACSL4), and their negative controls were purchased from OBio Technology (China). Transfection was performed using Lipofectamine 2000 (Invitrogen) as previously described [5]. Cells were then assessed for the overexpression or knockdown of FTX, podoplanin, FEN1, and ACSL4 using western blotting or qRT-PCR analysis.

### Laser capture microdissection

32 OSCC and corresponding adjacent normal tissue samples were prepared, and laser capture microdissection was performed. Using a microdissection microscope (Leica AS LMD, Leica) with a pulsed 337-nm UV laser, a trained pathologist performed two-step process for tissues collection. The RNA from independent malignant tissues was analyzed by qRT-PCR for comparison to normal samples.

### RNA extraction and qRT-PCR

Total RNA was extracted using TRIzol reagent (Invitrogen) according to the manufacturer’s instructions and was reverse transcribed into cDNA using a cDNA Reverse Transcription Kit (Takara). qRT-PCR was performed on a LightCycler 480 (Roche) with Fast Start Universal SYBR Green Master Mix (Roche) according to the manufacturer’s instructions. The relative mRNA expression was determined using the comparative Ct (ΔΔCt) method. The sequences of the primers are shown in Supplementary Table S1. All results were normalized to GAPDH or U6. All experiments were confirmed in three independent experiments.

### Microarray expression profiling

Exosomes derived from CAFs/Control and CAFs/sh-PDPN cells were subjected to a lncRNA microarray, and lncRNA sequencing was performed by RiboBio (China). We identified statistically significant differentially expressed lncRNAs using volcano plot filtering and calculated a *P* value using Student’s *t* test. The threshold for upregulated and downregulated genes was a fold change of 2.0 or greater and a *P* value of 0.05 or less. Finally, we performed hierarchical clustering to show the distinguishable lncRNA expression patterns among the samples.

### Functional assay

CAFs and OSCC cells used in the functional assays were transfected with the indicated plasmids, and stable colonies were selected. Functional assays, including proliferation, migration, invasion, and colony formation assays, were performed as previously described [5].

### In situ hybridization

In situ hybridization (ISH) was performed on 4-μm-thick paraffin sections using the RNAscope^®^ method to detect lncRNA FTX in OSCC and normal tissue as described previously [18]. The following probes were used (probe name, catalog number, GenBank accession number, number of probe pairs, and probe target region): Hs-*FTX*, Cat No. 1082731-C1, NR_028379.1, 10 pairs, nt 1066-2228. Sections were scanned and analyzed using an Apreio AT2 digital whole slide scanner (Leica, Wetzlar, Germany).

### Western blotting

Whole cells were collected and lysed in RIPA buffer (Sigma-Aldrich) containing protease inhibitors. Protein extracts were resolved by 10–12% SDS‒PAGE and transferred to PVDF membranes, and the signal was visualized using ECL Substrate (Millipore). The following primary antibodies were used: anti-podoplanin (ab236529, Abcam), anti-α-SMA (ab5694, Abcam), anti-GPX4 (ab125066, Abcam), anti-CD63 (ab134045, Abcam), anti-GM130 (ab52649, Abcam), anti-TSG101 (ab125011, Abcam), anti-TGF-β1 (ab215715, Abcam), anti-MMP2 (ab92536, Abcam), anti-MMP9 (ab76003, Abcam), anti-FEN1 (PA5-88244, Invitrogen), anti-TET2 (ab230358, Abcam), anti-ACSL4 (22401-1-AP, Proteintech), anti-SLC7A11 (ab307601, Abcam), anti-GAPDH (ab8245, Abcam).

### Exosome isolation and fluorescent labeling of exosomes

As in our previous study, the differential centrifugation method was used to isolate the exosomes from culture supernatants [5]. The retained exosomes were stored at −80 °C. Exosomes and OSCC cells were fluorescently labeled using PKH26 (Sigma-Aldrich) and Actin-Tracker Green (phalloidin-FITC, Beyotime Biotechnology), respectively. OSCC cells were incubated with labeled exosomes for 24 h. Confocal laser scanning microscopy (Zeiss) was used to visualize the endocytosis of exosomes by OSCC cells.

### Immunohistochemistry

Four-micrometer-thick serial sections were obtained, and immunostaining was performed as described in our previous study [19]. The primary antibody was anti-podoplanin (ab236529, Abcam), anti-Ki 67 (ab15580, Abcam), anti-ACSL4 (22401-1-AP, Proteintech), anti-SLC7A11 (ab307601, Abcam), and anti-GPX4 (ab125066, Abcam). The immunostaining results were analyzed by two independent pathologists who were blinded to the information of each patient. Quantitative analysis was performed as previously described [19].

### Immunofluorescence

Cells were fixed with 4% paraformaldehyde for 20 min and treated with 0.1% Triton X-100 for 5 min. The cells were then incubated with anti-human antibody against α-SMA (ab5694, Abcam), vimentin (ab20346), PDPN (ab236529, Abcam), and cytokeratin 14 (ab119695, Abcam) overnight at 4 °C, followed by incubation with antibodies conjugated with fluorescent Alexa Fluor 488 or rhodamine (Cell Signaling Technology) at 37 °C for 60 min. The immunofluorescence was visualized under a fluorescence microscope (LSM 5, Carl Zeiss, Germany).

### RNA‒protein pulldown assay

Full-length sense-FTX and antisense-FTX were transcribed using T7 MEGAscript kits and labeled using the Pierce RNA 3 End Desthiobiotinylation Kit (Thermo Fisher Scientific) in vitro. RNA pulldown assays were performed using the Pierce Magnetic RNA‒Protein Pull Down Kit (Thermo Fisher Scientific). Desthiobiotin-labeled RNA (50 pmol/L) was mixed with 50 mL of magnetic beads and then incubated with protein lysates from OSCC cells for 60 min at 4 °C with rotation. The beads were washed briefly four times and boiled in SDS buffer, and the RNA-binding proteins were detected by western blotting or mass spectrometry.

### RNA immunoprecipitation (RIP)

RNA immunoprecipitation (RIP) was conducted using the Magna Nuclear RIP (Cross-Linked) Nuclear RNA-Binding Protein Immunoprecipitation Kit (Millipore) according to the manufacturer’s protocol. Anti-FEN1 antibodies (PA5-88244, Invitrogen) were used for the RIP assay.

### Chromatin immunoprecipitation (ChIP)

Chromatin immunoprecipitation (ChIP) was performed using the ChIP Assay Kit (Beyotime) according to the manufacturer’s protocol. Briefly, cross-linked chromatin was sonicated into 200–1000 bp fragments. Chromatin was immunoprecipitated using anti-FEN1 antibodies (PA5-88244, Invitrogen). Normal human IgG (ab37415, Abcam) was used as an isotype control. Quantification of the immunoprecipitated DNA was performed using qPCR with SYBR Green Mix (Roche).

### Animal models

BALB/c nude mice (Beijing Vital River) at 4–6 weeks of age were used. To investigate the role of podoplanin-transfected CAFs in the tumorigenesis of OSCC cells, we subcutaneously injected SCC15 cells (5 × 10^6^ cells/body) and PDPN-overexpressing CAFs (5 × 10^6^ cells/body). To investigate the role of FEN1-transfected OSCC cells in tumorigenesis, we injected SCC15/FTX-OE cells (5 × 10^6^ cells/body), SCC15/Mock cells (5 × 10^6^ cells/body) and RSL-3 treated FEN1-transfected cells (5 × 10^6^ cells/body) subcutaneously. Mice were sacrificed 6 weeks after tumor inoculation or if they showed signs of distress. After sacrifice, tumors were dissected, collected, and weighed. Tumor tissues were used for immunohistochemistry study for anti-Ki67 (ab15580, Abcam), anti-ACSL4 (22401-1-AP, Proteintech), anti-SLC7A11 (ab307601, Abcam), and anti-GPX4 (ab125066, Abcam). Serial sections (4-μm thick) were cut, and hematoxylin and eosin (H&E) and immunostaining were performed, as described previously [19]. This study was approved by the Ethical Committee on Animal Research of Sun Yat-sen University (Approval number: SYSU-IACUC-2022-000261). All experimental procedures were performed according to national guidelines regarding the care and use of laboratory animals.

### Statistical analysis

All statistical analyses were performed using the Statistical Package for the Social Sciences version 20.0 software. For comparisons, the Student’s t test for paired data was used to compare mean values. ANOVA was used to analyze potential differences between two groups with continuous variables. All experiments were repeated at least three times with triplicates unless stated otherwise. All tests were twosided, and *P* values < 0.05 were considered to be statistically significant.

## Results

### Abundance of podoplanin expression in OSCC stroma

**Fig. 1:**
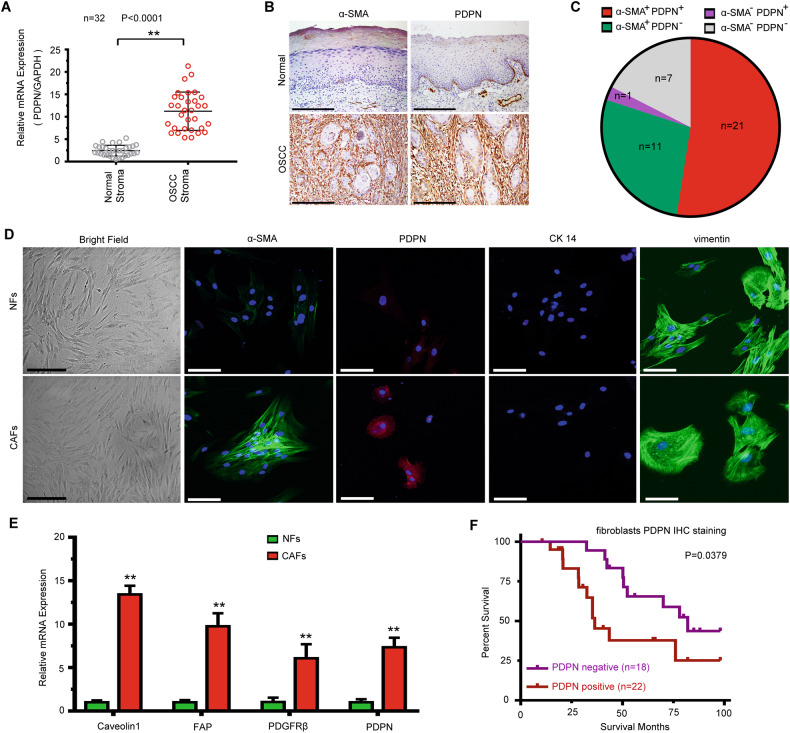
Podoplanin is overexpressed in OSCC stroma and is correlated with poor prognosis. **A** Gene expression levels of PDPN in normal and OSCC stroma (*n* = 32) using laser-captured microdissection and qRT-PCR. Student’s t test for two-group comparison: **P <0.01. **B** Immunohistochemical staining of PDPN and α-SMA in OSCC tissues and adjacent normal tissues (*n* = 40). Scale bar, 200 µm. **C** The proportion of different extent of PDPN and α-SMA expression in OSCC stroma. **D** Immunofluorescence staining identification of CAFs using vimentin, PDPN, cytokeratin 14, and α-SMA. Bar for morphological observation, 20 µm; bar for immunofluorescence staining, 100 µm. **E** Gene expression levels of activated fibroblasts markers in NFs and CAFs. Student’s t test for two-group comparison: **P <0.01. **F** Kaplan–Meier plots of PDPN expression in 40 cases of OSCC patients. Overall survival rate was performed by log-rank test. log-rank test: *P* < 0.05.

To investigate the expression of podoplanin (PDPN) in oral squamous cell carcinoma (OSCC) stroma, we analyzed the expression of PDPN in the stroma of 32 fresh human OSCC samples and corresponding adjacent normal tissues. As shown in Fig. 1A, OSCC stroma exhibited higher PDPN expression than normal stroma. It is well established that cancer-associated fibroblasts (CAFs), which are closely associated with OSCC cell invasion, are characterized by the expression of α-smooth muscle actin (α-SMA) [20]. We further evaluated the protein expression of PDPN and α-SMA in 40 paraffin-embedded OSCC tissues and adjacent normal tissues. IHC staining revealed that the stroma in 80.0% (32/40) of OSCC tissues expressed α-SMA and that the stroma in 55.0% (22/40) of OSCC tissues expressed PDPN (Fig. 1B). As shown in Fig. 1C, the stroma in 52.5% (21/40) of OSCC cases showed both α-SMA- and PDPN-positive expression. Next, we isolated primary normal fibroblasts (NFs) and CAFs from patients with OSCC who underwent tumor resection. We found that both CAFs and NFs were vimentin-positive and cytokeratin-negative, and that α-SMA and PDPN expression was much higher in CAFs than in NFs (Fig. 1D). As shown in Fig. 1E, the mRNA levels of PDPN and activated fibroblast markers, including Caveolin1, FAP, and PDGFRβ, were much higher in CAFs than in NFs. According to PDPN expression in the tumor stroma, we divided all OSCC patients into negative and positive PDPN expression groups. The results showed that higher PDPN expression in the stroma was significantly correlated with poor prognosis (Fig. 1F).

### PDPN^+^ CAFs increase tumor cell mobility and proliferation in vitro and in vivo

**Fig. 2:**
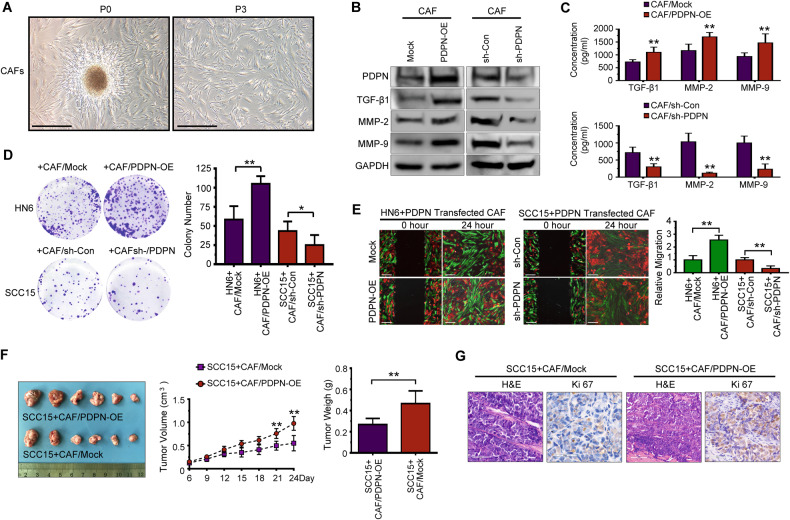
PDPN^+^ CAFs promote tumor cells mobility and proliferation in vitro and in vivo. **A** Representative morphology of CAFs derived from patients with OSCC. Scale bar, 200 μm. **B** Western blot analysis of PDPN, TGF-β1, MMP-2, and MMP-9 in PDPN-transfected CAFs. **C** Enzyme-linked immunosorbent assay (ELISA) analysis of TGF-β1, MMP2, and MMP9 expression in conditioned medium of PDPN-transfected CAFs. Student’s t test for two-group comparison: **P < 0.01. **D** The effect of PDPN-transfected CAFs indirect co-culture with OSCC cells on colony formation examined by colony formation assay. Student’s t test for two-group comparison: *P < 0.05; **P < 0.01. **E** The migration of OSCC cells direct co-cultured with PDPN-transfected CAFs was measured by wound healing assay. Bar, 200 μm. Student’s t test for two-group comparison: **P < 0.01. **F** SCC15 cells were co-injected with PPDN-overexpressed CAFs into nude mice (*n* = 6 for each group) subcutaneously. Tumors volume and weigh were evaluated. Student’s t test for two-group comparison: **P < 0.01. **G** Representative H&E and immunohistochemical staining images of Ki67 in SCC15 cells xenograft tumor tissue were shown. Scale bar, 100 μm.

The isolated primary CAFs showed a spindle-like morphology and could be grown for at least 12 passages (Fig. 2A). To determine the effect of PDPN on CAF behavior, we transfected CAFs with a lentiviral plasmid containing PDPN. Western blotting analysis indicated that PDPN overexpression increased the expression and secretion of TGF-β1, matrix metalloproteinase-2 (MMP2), and matrix metalloproteinase-9 (MMP9) (Fig. 2B, C). To clarify the function of PDPN^+^ CAFs in OSCC progression, we performed migration assays and a proliferation analysis involving cocultures of OSCC cells with PDPN-transfected CAFs. The results showed that PDPN overexpression in CAFs led to significantly elevated proliferation and migration of OSCC cells, and that PDPN knockdown led to decreased colony formation and migration of OSCC cells (Fig. 2D, E). To confirm the in vitro experimental observations, we established an OSCC tumor implantation model that involves the subcutaneous injection of SCC15 cells mixed with CAFs/PDPN-OE cells and CAFs/PDPN-Mock cells. Consistently, the CAF/PDPN-OE group had significantly increased tumor volume and weight compared with the CAF/PDPN-Mock group (Fig. 2F). Moreover, immunohistochemistry staining confirmed that PDPN-overexpressing CAFs caused increased Ki67 expression in SCC15 cells (Fig. 2G), which indicates that PDPN^+^ CAFs enhanced OSCC cell proliferation.

### PDPN^+^ CAF-derived exosomes transfer lncRNA FTX to OSCC cells

**Fig. 3:**
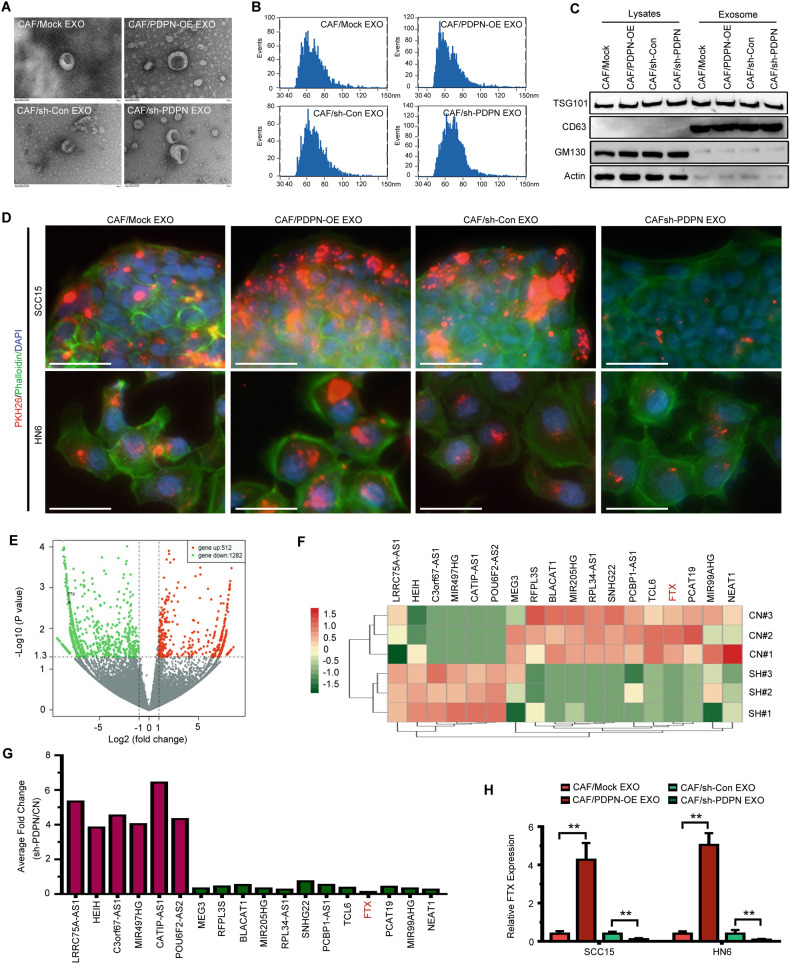
OSCC cells absorb exosomes derived from PDPN-transfected CAFs. **A** Transmission electron microscopy (TEM) showing exosomes isolated from PDPN-transfected CAFs conditioned medium. Scale bar, 100 nm. **B** The size distribution exosomes from PDPN-transfected CAFs examined by nanoparticle tracking analysis (NTA). **C** Western blot analysis of exosomes from PDPN-transfected CAFs. **D** OSCC cells absorbed PKH26-labeled exosomes (red) secreted by PDPN-transfected CAFs. Fluorescence microscopy was used to detect the phalloidin (green) and PKH26-labeled exosomes (red) fluorescent signals in OSCC cells. Bar, 100 μm. **E**, **F** Volcano plot (**E**) and heatmap diagram (**F**) showing differentially expressed lncRNAs between CAF/Control and CAF/sh-PDPN derived exosomes. **G** The expression of the selected lncRNAs in the CAF/Control and CAF/sh-PDPN derived exosomes was validated by qRT-PCR. **H** The expression of FTX in OSCC cells treated with PDPN-transfected CAFs derived exosomes was validated by qRT-PCR. Student’s t test for two-group comparison: **P < 0.01.

Previous studies have reported that exosomes are involved in the communication of CAFs and cancer cells [4, 21]. To further investigate the role of PDPN^+^ CAF-derived exosomes, we purified exosomes from the conditioned medium of PDPN-transfected CAFs. We found that all exosomes had a cup-shaped morphology and that their size ranged from 40 to 120 nm (Fig. 3A, B). Western blotting revealed the expression of the exosome markers TSG101 and CD63 but not GM130 and actin (Fig. 3C). OSCC cells were treated with isolated exosomes, and PKH-26-labeled exosomes were detected in both SCC15 and WSU-HN6 cells. As shown in Fig. 3D, exosomes from CAFs/PDPN-OE cells had an increased transfer capacity compared with those from CAFs/PDPN-Mock cells. Next, we evaluated the motility and proliferation of OSCC cells treated with the isolated exosomes. We found that exosomes derived from CAF/PDPN-OE cells increased the migration, invasiveness, and proliferation of OSCC cells (Fig. S1A–D). Consistently, OSCC cells treated with exosomes isolated from the CAFs/sh-Con group exhibited higher motility and proliferation ability than OSCC cells treated with exosomes isolated from the CAFs/sh-PDPN group (Fig. S1A–D). To confirm the effect of exosomes secreted from PDPN overexpressed CAFs, we treated the transfected CAFs with GW4869, an exosome inhibitor, in the co-culture system. We found that GW4869 treatment attenuated the elevated mobility and proliferation of OSCC cells promoted by PDPN^+^ CAFs co-culture (Fig. S1E, F). It has been reported that exosomal lncRNAs regulate the proliferation and motility of OSCC cells [22]. To determine the lncRNA profiles of exosomes derived from PDPN-transfected CAFs, we established PDPN-knockdown CAFs and performed a microarray analysis (Fig. 3E). We found eighteen differentially expressed lncRNAs (Fig. 3F), including upregulated and downregulated lncRNAs (fold change ≥2, *P* < 0.05), and we validated the microarray data by qRT-PCR (Fig. 3G). The microarray data and qRT-PCR results revealed that exosomal lncRNA FTX expression was decreased significantly in the PDPN-silenced group (Fig. 3E–G). Next, we evaluated the expression of FTX in exosome-treated OSCC cells. The results revealed that FTX expression in OSCC cells incubated with exosomes from CAF/PDPN-OE cells was much higher than that in OSCC cells incubated with exosomes from the control group (Fig. 3H).

### LncRNA FTX promotes OSCC cell motility by interacting with FEN1

**Fig. 4:**
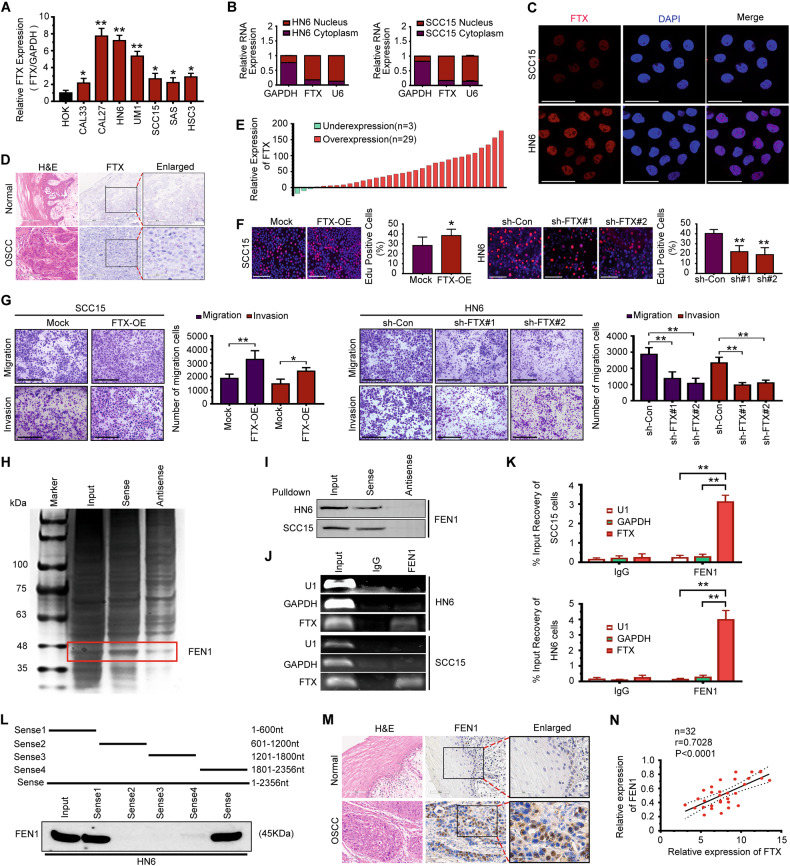
LncRNA FTX promotes OSCC cell motility and proliferation by interacting with FEN1. **A** qRT-PCR analysis was used to detect the expression of FTX in OSCC cell lines. Student’s t test for two-group comparison: *P < 0.05; **P < 0.01. **B** The expression of FTX in the subcellular fractions of WSU-HN6 and SCC15 cells were detected by qRT-PCR. U6 and GAPDH were used as nuclear and cytoplasmic markers, respectively. **C** The location of FTX (red) in WSU-HN6 and SCC15 cells were determined by FISH assay. **D** Representative ISH staining of FTX in OSCC and normal tissue sections. Small and large black frames indicate the original and magnified areas, respectively. Bar, 100 µm. **E** The expression level of FTX in OSCC patient tissue and adjacent normal tissue were detected by qRT-PCR (*n* = 32). **F** The effect of FTX transfection in the growth rate of both WSU-HN6 and SCC15 cells examined using EdU assays. Scale bar, 200 μm. Student’s t test for two-group comparison: *P < 0.05; **P < 0.01. **G** The effect of FTX transfection on the migration and invasion of SCC15 and WSU-HN6 cells was examined using the transwell assay. Scale bar, 200 μm. Student’s t test for two-group comparison: *P < 0.05; **P < 0.01. **H** Silver staining of biotinylated FTX-associated proteins. **I** The specific association of FTX with the FEN1 protein was validated through RNA pulldown, followed by western blotting. **J**, **K** PCR was used to measure the RNA enrichment in the RIP assay using the anti-FEN1 antibody in SCC15 and WSU-HN6 cells. Normal IgG was used as the nonspecific control antibody. U1 and GAPDH were used as negative controls. Student’s t test for two-group comparison: **P < 0.01. **L** Serial deletions of FTX were used in the RNA pulldown assays. **M** Representative H&E and immunohistochemical staining for FEN1 in paraffin-embedded OSCC and adjacent normal tissue sections. Small and large black frames indicate the original and magnified areas, respectively. Bar, 100 µm. **N** The correlation between FTX and FEN1 mRNA expression in fresh OSCC tissue samples (*n* = 32).

In this study, we selected FTX, which is encoded by a gene on chromosome Xq13.2 in the genome, for further study. A schematic of the genomic locus of FTX is shown in Supplemental Fig. 2A. Plasmids cloned with full-length FTX were constructed and then transfected into HEK 293T cells. Western blot analysis showed that FTX has no coding capability (Fig. S2B). We used online tools to determine the coding capability of the FTX gene and found that the capacity of FTX for gene coding is low (Fig. S2C). LncRNA FTX has been shown to regulate cancer cell proliferation, motility, and aberrant metabolism in many cancers, including colorectal cancer (CRC), hepatocellular carcinoma (HCC), and renal cell carcinoma (RCC) [23]. However, its role in the progression of OSCC still unclear. To investigate the biological function of FTX, we analyzed the endogenous expression of FTX in OSCC cell lines. The expression of FTX in OSCC cell lines was significantly higher compared with that in human oral keratinocyte (HOK) cells (Fig. 4A). The results of nuclear/cytoplasmic RNA fractionation from the subcellular distribution assay confirmed that FTX was primarily located in nucleus (Fig. 4B), which was further confirmed by fluorescence in situ hybridization (FISH) analysis (Fig. 4C). Next, we used ISH staining to detect FTX expression in paraffin-embedded OSCC tissue sections (*n* = 40), and we found that FTX was significantly overexpressed in OSCC tissues compared with normal epithelium (Fig. 4D). Moreover, we found that the expression of FTX was increased in 29 of 32 (90.6%) fresh tumor samples compared with adjacent normal tissues (Fig. 4E). Then, we divided all OSCC patients (*n* = 40) into high and low FTX expression groups. The results showed that higher expression of FTX was significantly correlated with poor differentiation (*P* = 0.048), lymph node metastasis (*P* = 0.041), and clinical stage (*P* = 0.034) (Table 1). Survival rate analysis also showed that higher FTX expression was significantly correlated with shorter survival (Fig. S3). Next, we overexpressed FTX in SCC15 cells and knocked down FTX in WSU-HN6 cells by lentiviral plasmid transfection. The transfection efficiency was confirmed by qRT-PCR (Fig. S4). Significantly higher proliferation rates were observed in FTX-overexpressing SCC15 cells than in control cells (Fig. 4F). In contrast, depletion of FTX decreased the proliferation rate of WSU-HN6 cells (Fig. 4F). As shown in Fig. 4G, FTX overexpression upregulated the migration and invasiveness of SCC15 cells, and FTX depletion dramatically decreased the motility of WSU-HN6 cells.

**Table 1:** Correlation between LncRNA FTX expression and clinicopathological parameters in oral squamous cell carcinomas.

Features	Number	LncRNA FTX expression level		*P*
		Low	High	
*Age (years)*				
≤60	13	5	8	0.720
>60	27	12	15	
*Gender*				
Male	22	10	12	0.949
Female	18	8	10	
*Location*				
Tongue	12	5	7	0.641
Gingival	6	3	3	
Buccal	10	6	4	
Others	12	4	8	
*Differentiation*				
Well	10	7	3	0.048*
Moderate	20	5	15	
Poor	10	3	7	
*Tumor size*				
T1–T2	30	17	13	0.144
T3–T4	10	3	7	
*Lymph node*				
pN^−^	33	23	10	0.041*
pN^+^	7	2	5	
*Clinical stage*				
I–II	34	26	8	0.034*
III–IV	6	2	4	

Data were analyzed using the Chi-squared test.

*P < 0.05.

To investigate the mechanism of the tumorigenic functions of FTX, we hypothesized that FTX might bind to a protein that mediates these effects. To identify such a protein, we performed an RNA pull-down assay. We found that FTX was specifically bound to 35–48 kDa protein bands (Fig. 4H), and mass spectrometry identified the primary protein in the FTX interaction as flap endonuclease-1 (FEN1) (Fig. 4H). Other potential proteins that interact with FTX are shown in Supplementary Table S2. To confirm that FTX binds specifically to FEN1, we repeated the pulldown assay with biotinylated FEN1 and probed for FTX using immunoblot analysis. We obtained a similar result that FTX was specifically bound to FEN1 (Fig. 4I). RIP-qPCR showed that FTX was significantly enriched with the anti-FEN1 antibody compared with the control (Fig. 4J, K). In addition, a serial deletion analysis revealed that the 1–600 nt region in the 5’ terminal end of the FTX transcript was critical for its interaction with FEN1 (Fig. 4L). To investigate the role of FEN1 in OSCC progression, we detected the expression of FEN1 in 40 paraffin-embedded OSCC tissue samples and adjacent normal tissues by IHC. The results showed that OSCC samples displayed higher expression of FEN1 than normal samples (Fig. 4M). To further confirm the correlation of FTX and FEN1 expression, qRT-PCR was performed in 32 fresh OSCC tissues and adjacent normal tissues. FTX overexpression was significantly correlated with FEN1 upregulation (Fig. 4N).

### LncRNA FTX recruits TET2 to the FEN1 promoter region and causes DNA demethylation of FEN1

**Fig. 5:**
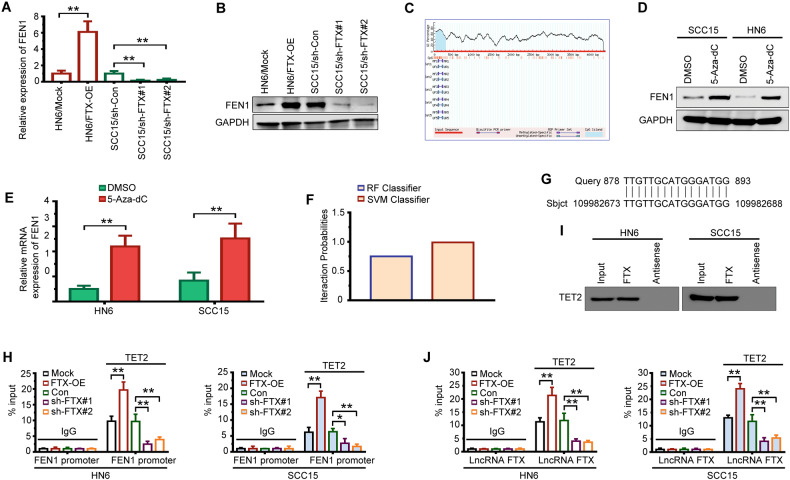
LncRNA FTX regulates FNE1 expression through TET2-mediated FEN1 promoter demethylation. **A**, **B** The effect of lncRNA FTX transfection on FEN1 expression detected by qRT-PCR (**A**) and western blotting (**B**). Student’s t test for two-group comparison: **P < 0.01. **C** Prediction of CpG island enrichment in FEN1 promoter region by the MethPrimer website. **D**, **E** The expression of FEN1 in WSU-HN6 and SCC15 cells treated with 5-Aza-dC detected by western blotting (**D**) and qRT-PCR (**E**). Student’s t test for two-group comparison: **P < 0.01. **F** RPIseq databank predicts DNA demethylase TET2 are relevant to FTX using RF classifier and SVM classifier (RF > 0.5 & SVM > 0.5). **G** Potential binding sites for FTX within FEN1 promoter region verified using BLAST. **H** The enrichment of TET2 in FEN1 promoter region assessed by CHIP assay, and quantified by qRT-PCR. Student’s t test for two-group comparison: *P < 0.05; **P < 0.01. **I** The binding relationship between FTX and TET2 examined by RNA pull-down. **J** The enrichment of FTX by TET2 assessed by RIP assay, and quantified by qRT-PCR. Student’s t test for two-group comparison: **P < 0.01.

To further confirm the interaction between FTX and FEN1, we overexpressed or silenced FTX in OSCC cells. We found that FEN1 expression in WSU-HN6 cells was increased dramatically after upregulation of FTX and that FEN1 expression was remarkably decreased in SCC15 cells after FTX was silenced (Fig. 5A, B). Accumulating evidence has revealed that the subcellular localization of lncRNAs is strongly associated with their functions and mechanisms [21, 24]. Since FTX was mainly located in nucleus (Fig. 4B, C), we speculated that FTX may regulate FEN1 expression at transcriptional level. To further investigate the way in which FTX regulates FEN1 expression, we identified one CpG site in the promoter region of FEN1 using MethPrimer (http://www.urogene.org/cgi-bin/methprimer/methprimer.cgi) (Fig. 5C). We treated OSCC cells with the methyltransferase inhibitor 5-Aza-dC. As expected, the expression of FEN1 was notably increased in both WSU-HN6 and SCC15 cells (Fig. 5D, E). By exploring the RPIseq databank (http://pridb.gdcb.iastate.edu/RPISeq/), we found that the DNA demethylase ten-eleven translocation-2 (TET2) was a binding partner of FTX (Fig. 5F). In addition, online comparison using the BLAST tool showed that FTX may bind to the FEN1 promoter in an RNA-DNA manner (Fig. 5G). These findings strongly suggested that FTX might activate FEN1 expression by TET2 recruitment and FEN1 promoter demethylation. To study the enrichment of TET2 in the promoter of FEN1, we conducted a ChIP assay after overexpressing and silencing FTX in OSCC cells. As shown in Fig. 5H, we found that more TET2 was enriched in the FEN1 promoter in the FTX-overexpressing group, while the opposite effect was observed in the FTX-silenced group. The RNA pull-down assay further verified that FTX recruited TET2 (Fig. 5I). Furthermore, RIP assays revealed that the enrichment of FTX by TET2 increased after FTX was overexpressed, whereas it declined after FTX was silenced (Fig. 5J).

### FEN1 overexpression promotes the motility of OSCC cells by regulating ferroptosis

**Fig. 6:**
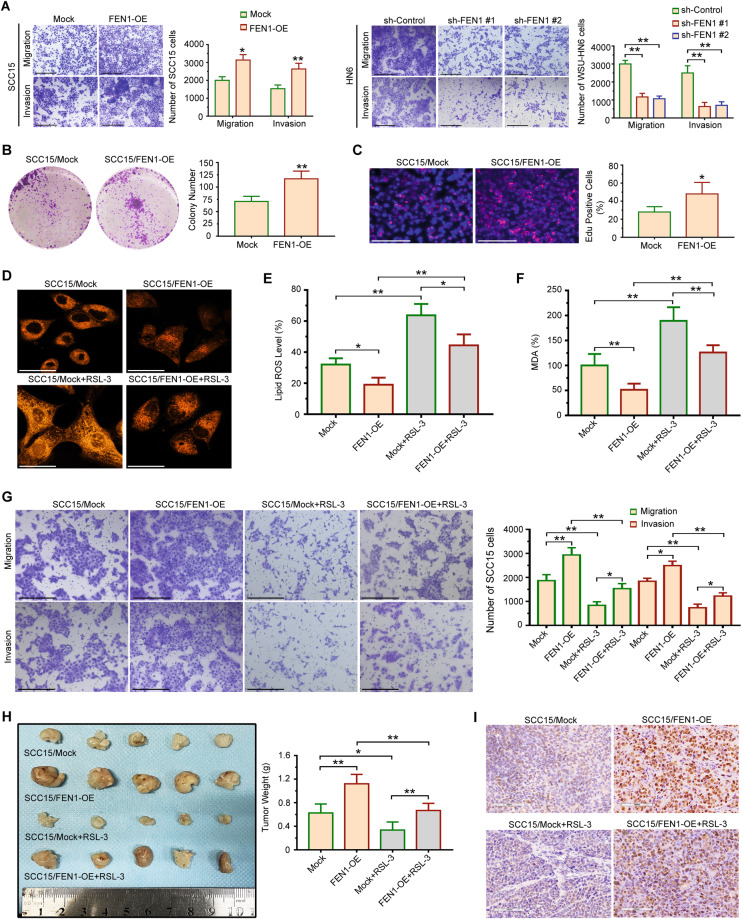
FEN1 overexpression promotes OSCC cells motility and proliferation by regulating ferroptosis in vitro and in vivo. **A** The effect of FEN1 transfection on the migration and invasion of SCC15 and WSU-HN6 cells was examined using the transwell assay. Scale bar, 200 μm. Student’s t test for two-group comparison: *P < 0.05; **P < 0.01. **B**, **C** The effect of FEN1 overexpression on OSCC cells growth examined using colony formation assay (**B**) and EdU assays (**C**). Scale bar, 200 μm. Student’s t test for two-group comparison: *P < 0.05; **P < 0.01. **D** The effect of adding RSL-3 on the level of intracellular iron in FEN1 overexpressed OSCC cells was determined using FerroOrange. Scale bar, 50 μm. **E** The effect of adding RSL-3 on the level of lipid ROS in FEN1 overexpressed OSCC cells was measured by ROS assay kit. One-way ANOVA for multi-group comparisons: *P < 0.05; **P < 0.01. **F** The effect of adding RSL-3 on level of Malondialdehyde (MDA) in FEN1 overexpressed OSCC cells was evaluated by a lipid peroxidation MDA assay. One-way ANOVA for multi-group comparisons: **P < 0.01. **G** Transwell assay was used to determine the effect of RSL-3 treatment on migration and invasion of FEN1 overexpressed SCC15 cells. Scale bar, 200 μm. One-way ANOVA for multi-group comparisons: *P < 0.05; **P < 0.01. **H** SCC15/Mock, SCC15/FEN1-OE, SCC15/Mock+RSL-3, and SCC15/FEN1-OE + RSL-3 cells were injected into nude mice (*n* = 5 for each group) subcutaneously. Tumors weigh was evaluated. One-way ANOVA for multi-group comparisons: *P < 0.05; **P < 0.01. **I** Representative immunohistochemical staining images of Ki 67 in xenografts were shown. Scale bar, 100 μm.

To identify the role of FEN1 in the invasion of OSCC cells, we transfected FEN1 gene into SCC15 cells. We found that higher FEN1 expression was found in SCC15/FEN1-OE cells compared with SCC15/Mock cells (Fig. S5A). As shown in Fig. 6A, forced FEN1 expression increased the invasiveness of OSCC cells. To further confirm the relationship between FEN1 expression and cell invasiveness, shRNA strategy was used to knockdown FEN1 in WSU-HN6 cells. The result demonstrated FEN1 shRNA strategy effectively suppressed FEN1 expression in WSU-HN6 cells (Fig. S5B), and the invasiveness of WSU-HN6 cells decreased significantly after FEN1 was knockdown (Fig. 6A). In addition, FEN1 overexpression promoted the colony formation (Fig. 6B), and proliferation rates (Fig. 6C). Ubellacker et al. reported that ferroptosis regulates the malignant behaviors of melanoma cells [25]. Next, we used an Fe^2+^ iron probe known as FerroOrange to evaluate intracellular iron levels in OSCC cells. As shown in Fig. 6D, FEN1 overexpression decreased the Fe^2+^ level in SCC15/FEN1-OE cells, compared to SCC15/Mock cells. Furthermore, ferroptosis-inducing agents, RSL-3, increased the Fe^2+^ level in both SCC15/Mock and SCC15/FEN1-OE cells (Fig. 6D). Lipid ROS accumulation and the levels of malondialdehyde (MDA) were detected in FEN1-overexpressed SCC15 cells treated with RSL-3. The results showed that decreased ROS production (Fig. 6E) and levels of MDA (Fig. 6F) in FEN1 overexpressed group was significantly upregulated by RSL-3 treatment. The combined biological effect of FEN1 transfection and RSL-3 treatment was detected by transwell assay. The migration and invasion ability of SCC15 cells upregulated by FEN1 overexpression could be markedly attenuated with treatment of RSL-3 (Fig. 6G). To further confirm our in vitro findings, we performed an in vivo tumorigenesis experiment in nude mice. The results showed that FEN1 overexpression significantly increased tumor weights compared with the control group (Fig. 6H). In addition, RSL-3 treatment suppressed the tumorigenesis potential of FEN1-overexpressed SCC15 cells (Fig. 6H). Moreover, immunohistochemistry staining confirmed that FEN1 overexpression caused increased Ki67 expression in SCC15 cells (Fig. 6I), and RSL-3 treatment suppressed Ki67 expression in both SCC15/Mock and SCC15/FEN1-OE cells (Fig. 6I). Thus, FTX/FEN1 axis might promote OSCC cells motility by inhibiting ferroptosis.

### ACSL4 depletion aggravates FEN1 induced promotion of motility of OSCC cells

**Fig. 7:**
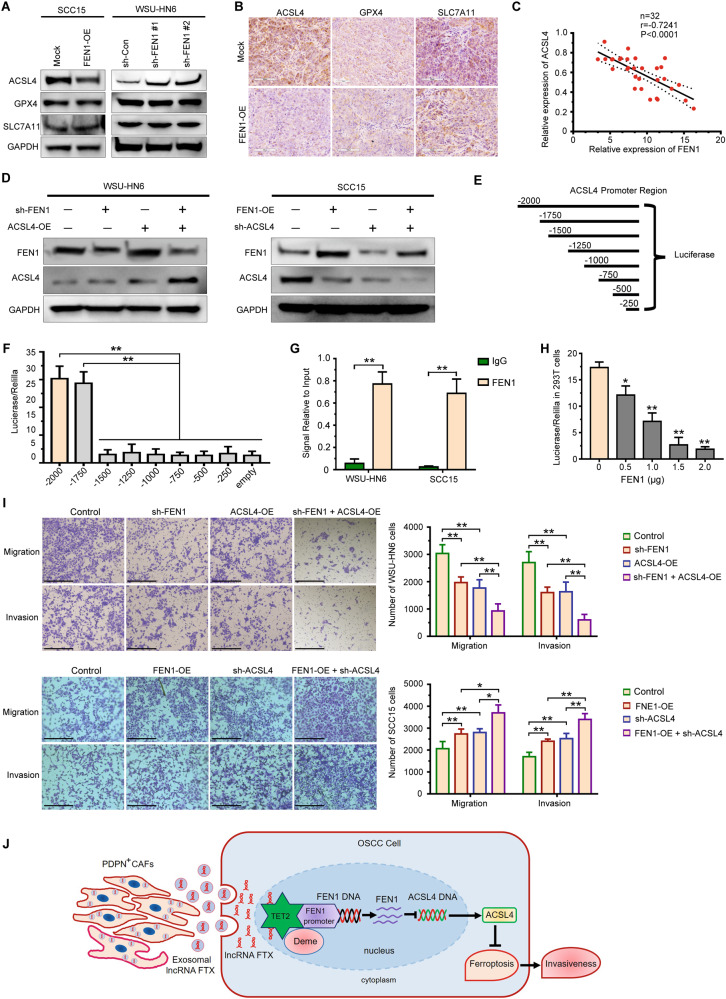
FEN1 expression is negatively correlated with ACSL4 expression in OSCC. **A** The expression of ferroptosis-related markers expression in FEN1-transfected OSCC cell lines detected by western blotting. **B** Representative immunohistochemical staining images of GPX4, SLC7A11, and ACSL4 in xenografts were shown. Scale bar, 100 μm. **C** The correlation between ACSL4 and FEN1 mRNA expression in fresh OSCC tissue samples (*n* = 32). **D** The expression of FEN1 and ACSL4 in OSCC cells transfected with FEN1 and ACSL4 plasmid was measured by western blotting. **E**, **F** Schematic view of the reporter constructs containing different intervals of the ACSL4 regulatory region, generated for the mapping of the FEN1 responsive region (**E**). Luciferase activity was measured 48 h post-transfection (**F**). one-way ANOVA for multi-group comparisons: **P < 0.01. **G** ChIP-qPCR assay of FEN1 and normal IgG in SCC15 and WSU-HN6 cells. Student’s t test for two-group comparison: **P < 0.01. **H** Luciferase assay of increasing amount of FEN1 on the luciferase activity of pGL3-Basic and pGL3-ACSL4 plasmid in 293 T cells. Student’s t test for two-group comparison: *P < 0.05; **P < 0.01. **I** Transwell assay was used to determine the effect of a FEN1 and ACSL4 plasmid transfection on migration and invasion of OSCC cells. Scale bar, 200 μm. one-way ANOVA for multi-group comparisons: *P < 0.05; **P < 0.01. **J** A proposed model illustrating the role of PDPN^+^ CAFs derived exosomal lncRNA FTX in regulating motility of OSCC cells by inhibiting ferroptosis through FTX/FEN1/ACSL4 signaling cascade.

We analyzed the expression of several key factors in pathways of ferroptosis, including SLC7A11, GPX4, and ACSL4. We found that FEN1 overexpression significantly inhibited ACSL4 levels in SCC15 cells, and that in WSU-HN6 cells with FEN1 stably knockdown was increased (Fig. 7A). However, FEN1 transfection did not affect the expression of SLC7A11 and GPX4 (Fig. 7A). Similar results can be observed by IHC staining of ACSL4, GPX4, and SLC7A11 in subcutaneous tumor (Fig. 7B). The correlation between FEN1 and ACSL4 was further confirmed in 32 clinical OSCC tissues and adjacent normal tissues. ACSL4 expression was progressively reduced with FEN1 overexpression (Fig. 7C). Next, we performed ACSL4 overexpression in FEN1-silenced WSU-HN6 cells, and ACSL4 knockdown was also conducted in FEN1-overexpressed SCC15 cells. We found that the overexpression of FEN1 leads to downregulation of ACSL4 expression in OSCC cell lines (Fig. 7D). The correlation between FEN1 and ACSL4 in OSCC cells prompted us to identify the underlying mechanism.

To identify putative FEN1 responsive elements within the promoter region of the ACSL4 gene, sequential deletions of the 5’ flanking region were generated and cloned in front of the luciferase gene in a pGL3 basic vector (Fig. 7E). Transfection of a vector, containing 1750 nucleotides of the ACSL4 regulative region, gave similar luciferase activity as cells transfected with full-length ACSL4–2000. A further deletion of 250 bp resulted, however, in a dramatic reduction in luciferase activity, suggesting that the small interval within −1750 and 1500 bp contains particularly effective FEN1 responsive element(s) (Fig. 7F). As shown in Fig. 7G, one target segment of the promoter region of ACSL4 was notably enriched in chromatin precipitated with an anti-FEN1 antibody in both WSU-HN6 and SCC15 cells more than anti-IgG, which indicates that FEN1 could suppress ACSL4 expression transcriptionally. Furthermore, we transfected the pGL3-ACSL4 reporter into 293T cells and treated with increasing dose of pCDH-FLAG-FEN1, and found that the luciferase activity was considerably attenuated (Fig. 7H). The data collectively showed that FEN1 downregulated ACSL4 expression by binding to the site (−1750/−1500) in the ACSL4 promoter region in OSCC cells.

The combined biologic effects of FEN1 and ACSL4 transfection was investigated by transwell assay. After ACSL4 overexpression, the migrated and invasive capacity of WSU-HN6 cells suppressed by FEN1 depletion could be aggravated. In addition, sh-ACSL4 introduction notably promoted the migration and invasion of FEN1-overexpressed SCC15 cells (Fig. 7I). Taken together, these results confirmed that ACSL4 depletion could aggravate the FEN1 induced promotion of motility of OSCC.

## Discussion

Previous studies have indicated that CAFs are heterogeneous and specific CAF subsets provide a supporting niche for sustaining the malignancy of cancer cells [26, 27]. Here, we found that podoplanin-positive cancer-associated fibroblasts (PDPN^+^ CAFs) is a distinct subtype of CAFs in tumor microenvironment and mediated the progression of oral squamous cell carcinoma (OSCC). Mechanically, we demonstrated that PDPN^+^ CAFs provide a constant source of the paracrine exosomal lncRNA FTX, and PDPN^+^ CAFs promote aggressiveness of OSCC cells by inhibiting ferroptosis through FTX/FEN1/ACSL4 signaling cascade.

In the tumor microenvironment, the function of PDPN^+^ CAFs remains controversial. It has been demonstrated that PDPN-expressing CAFs have an inhibitory effect on the proliferation of small cell lung cancer (SCLC) cells [28]. However, PDPN-expressing CAFs enhance the progression of pancreatic invasive ductal carcinoma (IDC) [29] and lung adenocarcinoma [17]. Consistently, we found that PDPN expression in CAFs was significantly associated with the prognosis of OSCC patients and that PDPN^+^ CAFs facilitated the motility of OSCC cells both in vitro and in vivo. Our data revealed that PDPN^+^ CAFs may be defined as a distinct CAF subset that promotes OSCC progression.

Extensive evidences have illustrated that exosomes mediate the horizontal transfer of information to specific recipient cells by delivering lncRNAs [6, 30, 31]. Currently, the contribution of exosomal lncRNAs to OSCC progression remains largely unclear. Our findings indicated that PDPN^+^ CAFs-secreted lncRNA FTX-enriched exosomes promote the proliferation and motility of OSCC cells. To confirm a precise causal relationship between FTX transfection and OSCC cell invasiveness, we overexpressed full-length FTX in SCC15 cells and knocked down FTX in WSU-HN6 cells using lentiviral plasmid transfection. Our data demonstrated that FTX promotes the proliferation and motility of OSCC cells both in vitro and in vivo.

The biological function of lncRNAs is largely dependent on their subcellular localization [32]. It has been demonstrated that FTX is distributed in the colorectal cancer (CRC) cell nucleus and cytoplasm and drives the malignant progression of CRC by regulating miR-214-5p/JAG1 axis [33]. However, recent studies have revealed that RNA-binding proteins play a crucial role in the lncRNA regulatory network. In colorectal cancer, FEZF1-AS1/PKM2 serves as a lncRNA/protein complex that is associated with cancer cell proliferation and metastasis by activating STAT3 signaling [34]. Flap endonuclease 1 (FEN1), a structure-specific nuclease, removes the RNA primers during Okazaki fragment maturation in DNA synthesis and is inducible during cell proliferation [35]. FEN1 overexpression has been shown to be associated with aggressive behavior and poor survival in different tumors, including breast cancer, ovarian cancer, and non-small cell lung cancer [36, 37]. We demonstrated that FTX is preferentially localized in the nucleus of OSCC cells and that FTX interacts with FEN1. It is well established that lncRNAs play roles in the development and progression of tumorigenesis via epigenetic modification, transcriptional regulation, and posttranscriptional regulation [38]. For instance, lncRNA HOTAIRM1 facilitates glioblastoma multiforme (GBM) progression by mediating H3K9 and H3K27 histone demethylation and reduces DNA methylation levels by sequestering DNA methyltransferases away from the TSS of the HOXA1 gene [39]. Ten-Eleven Translocation-2 (TET2) demonstrates functionality as a DNA demethylase that can convert 5-methyl-cytosine to 5-hydroxymethyl-cytosine [40]. Meng et al. reported that LINC-PINT mediates the proliferation and invasion of thyroid cancer cell via TET2 [41]. In our present study, we identified one CpG site in the FEN1 promoter region and revealed that FTX promotes the demethylation of FEN1 and promotes its expression by recruiting TET2 to the FEN1 promoter region.

Ferroptosis has been characterized as the accumulation of lipid peroxidation products in a cellular iron-dependent manner [42]. Recent studies have indicated that the overexpression of ferroptosis-related lncRNAs effectively promotes tumor progression [43, 44]. LncRNA PVT1 regulates ferroptosis through miR-214-mediated TFR1 and p53 expression [43]. However, the potential roles of FTX/FEN1 axis in regulating ferroptosis in OSCC cells are still unknown. In our present study, a series of phenotypes and ferroptosis-related factors were detected after treatment with RSL-3 (a ferroptosis activators) in vitro and in vivo. A key finding of the present study revealed that decreased ROS production and levels of MDA in FEN1 overexpressed group was significantly upregulated by RSL-3 treatment. More importantly, RSL-3 treatment suppressed the motility and tumorigenesis potential of SCC15/FEN1-OE cells compared to the SCC15/Mock cells. To sum up, our findings uncovered that FTX/FEN1 axis promotes tumor aggressiveness via suppressing ferroptosis in OSCC cell lines. ACSL4 belongs to long-chain fatty acyl CoA synthetase family (ACSLs) and catalyzes the acetylation of long chain polyunsaturated fatty acids to produce lipid peroxides [45]. Many studies have demonstrated that highly expressed ACSL4 increases cellular sensitivity to ferroptosis [46, 47]. In our study, ACSL4 was ultimately screened out and confirmed to participate in the motility promotion induced by FEN1 overexpression. Furthermore, FEN1 could bound to promoter region of ACSL4 and then inhibit ferroptosis in OSCC cells.

In conclusion, our study showed for the first time that a distinct cancer-promoting CAF subset, PDPN^+^ CAFs, secrete exosomal lncRNA FTX in the tumor microenvironment. PDPN^+^ CAFs promote the aggressiveness by inhibiting ferroptosis of OSCC cells via the FTX/FEN1/ACSL4 signaling cascade (Fig. 7J). Our data reveal a new direction for understanding the oncogenic roles of CAFs in OSCC progression and may provide an attractive treatment strategy for OSCC patients.

### Supplementary information

Figure S1

Figure S2

Figure S3

Figure S4

Figure S5

Supplementary figure legends

Supplementary Table 1

Supplementary Table 2

Original western blotting Data File

Reproducibility checklist

## Data Availability

The authors confirm that all data generated or analyzed during this study are available.
