# Supplementary figures and images for: PDPN+ CAFs facilitate the motility of OSCC cells by inhibiting ferroptosis via transferring exosomal lncRNA FTX
Source: Cell Death Dis. 2023 Nov 22;14(11):759. doi: 10.1038/s41419-023-06280-3 (PMC10665425; doi:10.1038/s41419-023-06280-3)

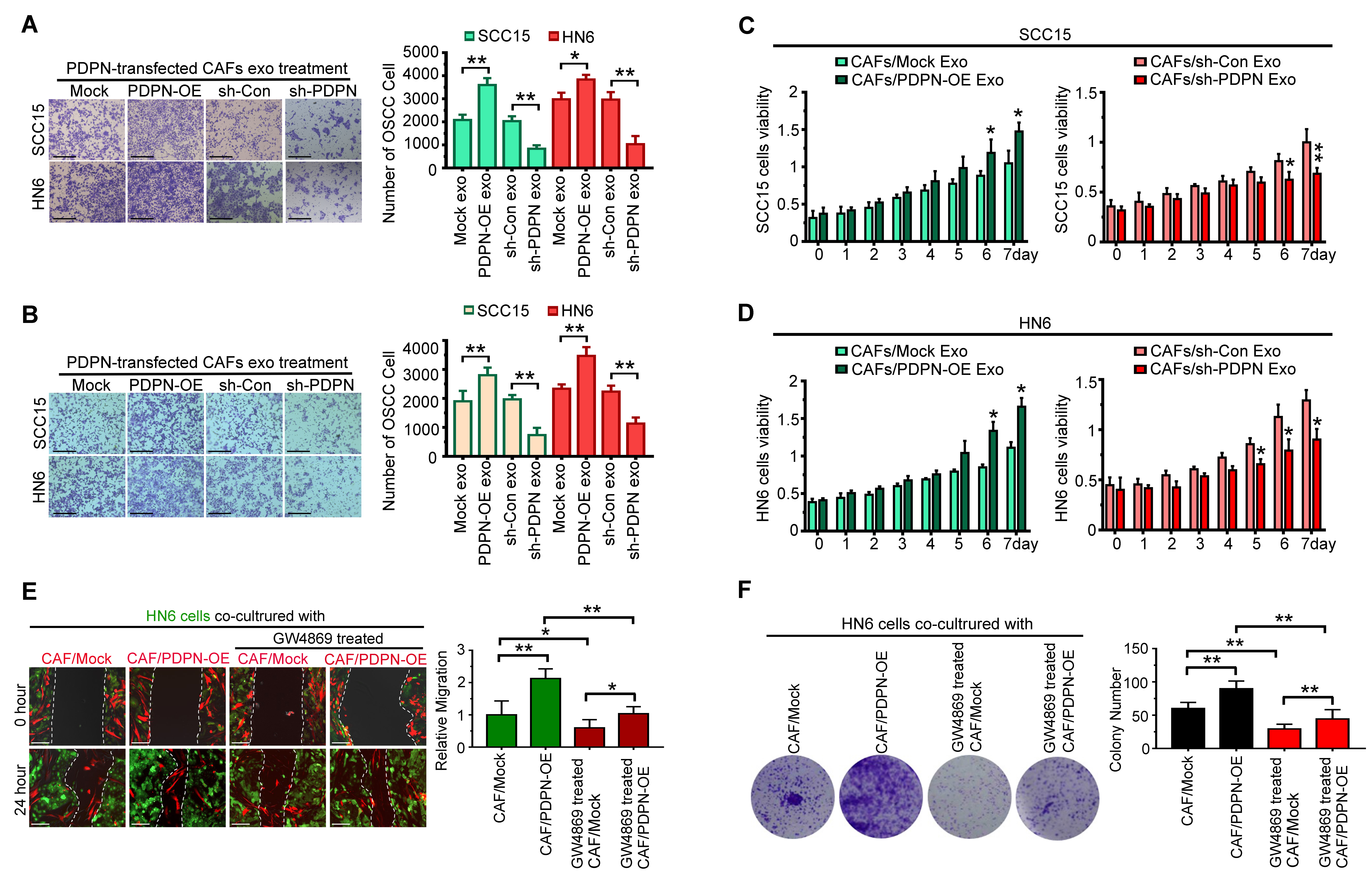

Supplement: Supplementary file 1 — Figure S1 [file 41419_2023_6280_MOESM1_ESM.jpg]

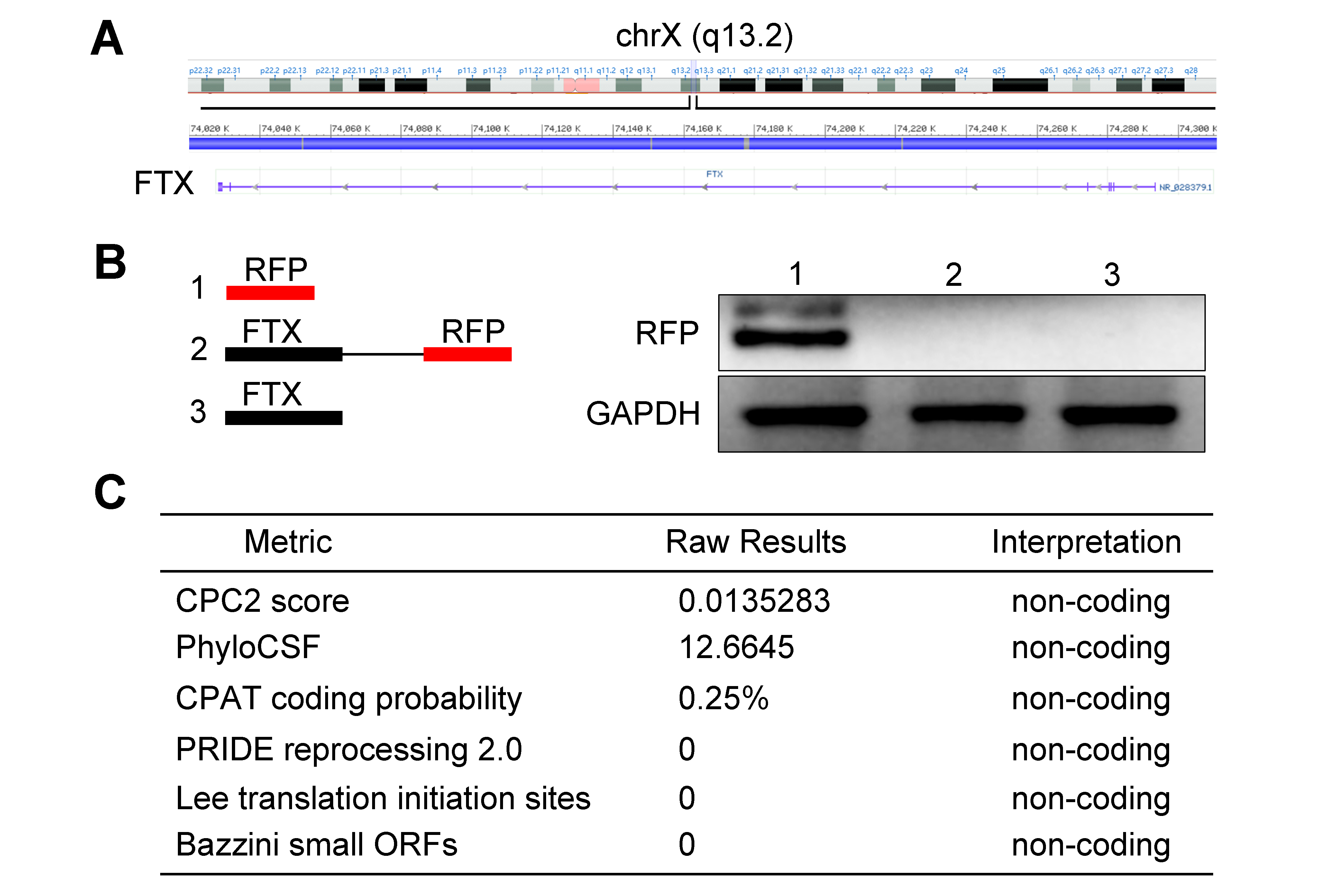

Supplement: Supplementary file 2 — Figure S2 [file 41419_2023_6280_MOESM2_ESM.jpg]

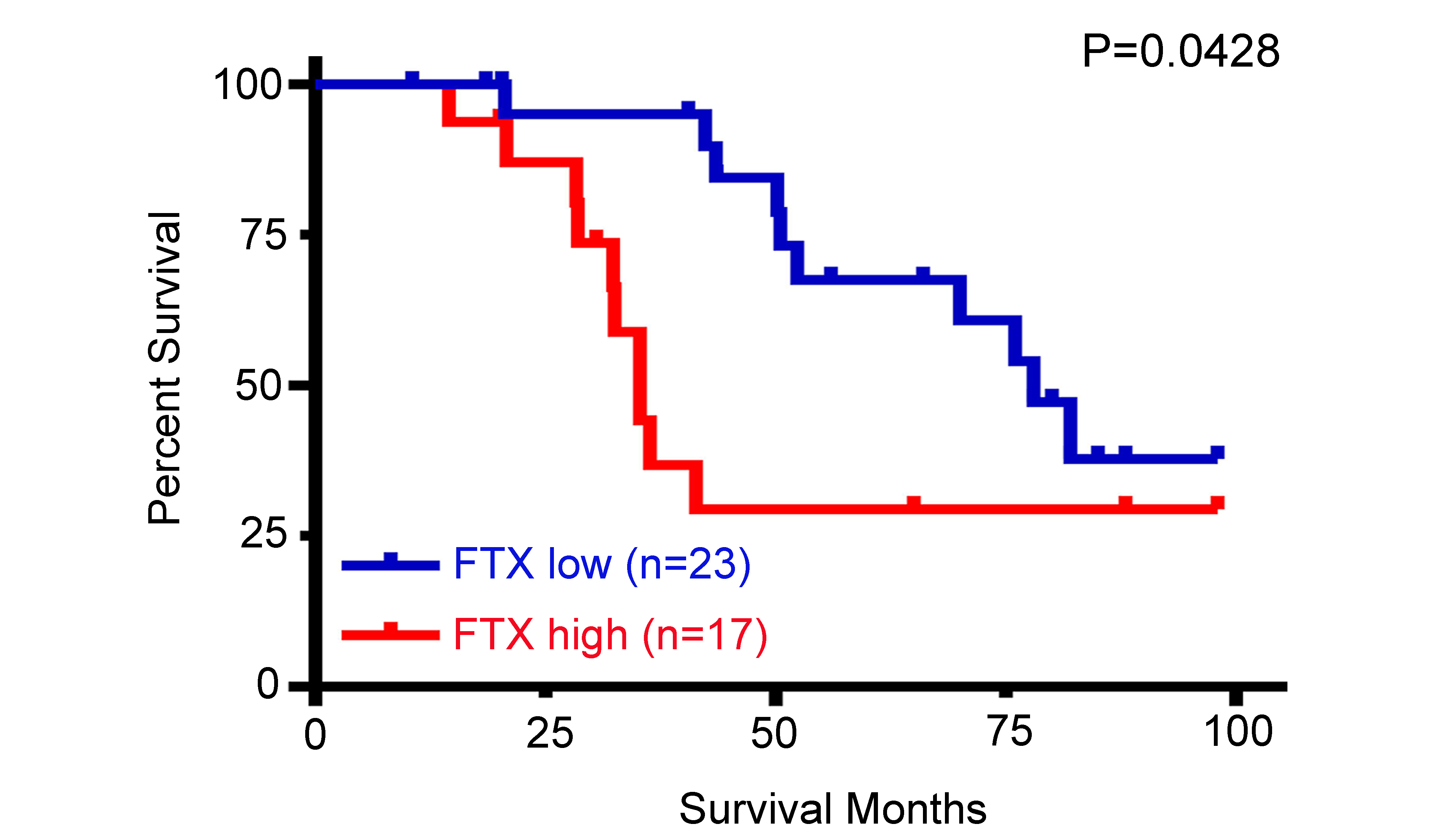

Supplement: Supplementary file 3 — Figure S3 [file 41419_2023_6280_MOESM3_ESM.jpg]

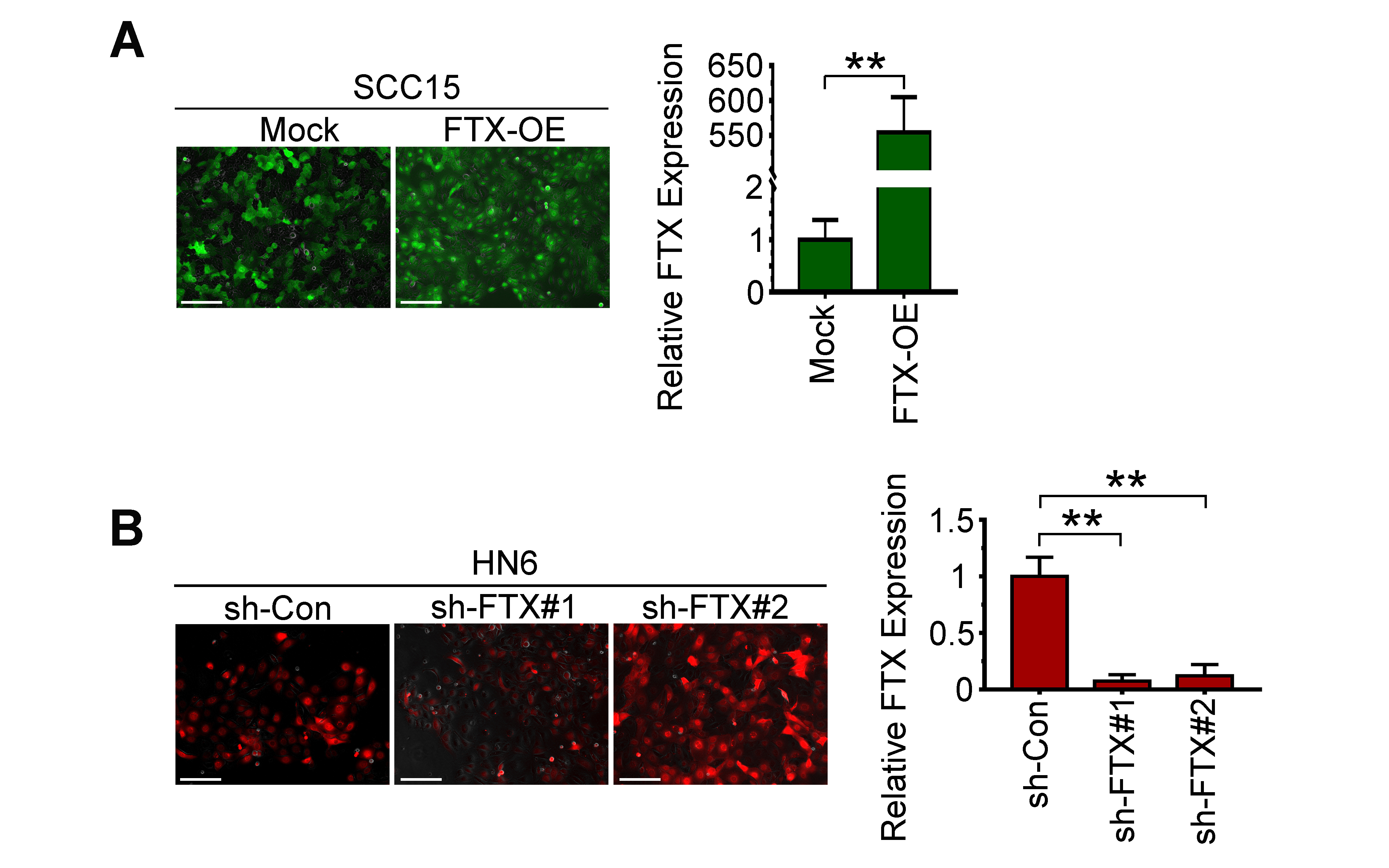

Supplement: Supplementary file 4 — Figure S4 [file 41419_2023_6280_MOESM4_ESM.jpg]

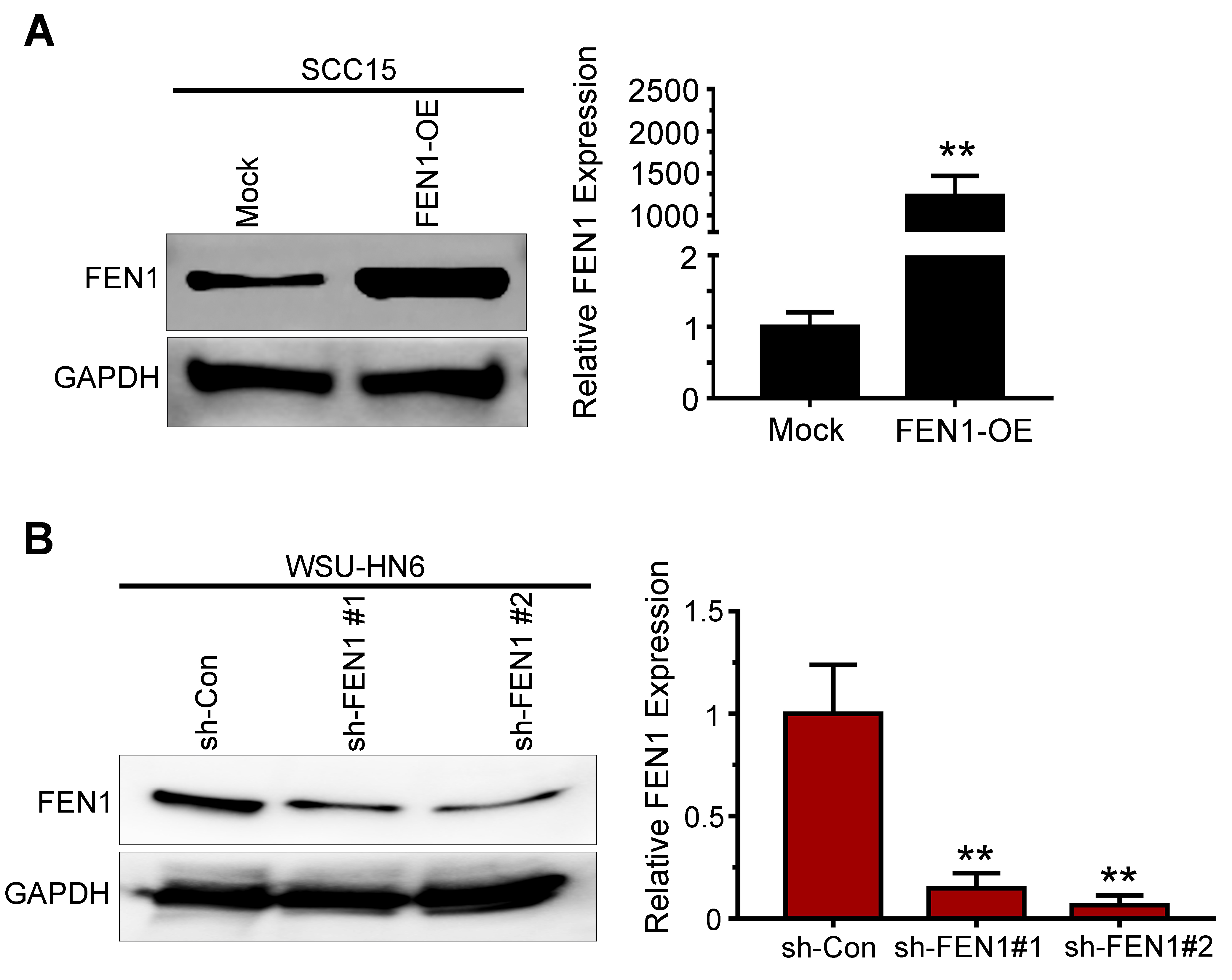

Supplement: Supplementary file 5 — Figure S5 [file 41419_2023_6280_MOESM5_ESM.jpg]
