# Supplementary material for: PDPN+ CAFs facilitate the motility of OSCC cells by inhibiting ferroptosis via transferring exosomal lncRNA FTX
Source: Cell Death Dis. 2023 Nov 22;14(11):759. doi: 10.1038/s41419-023-06280-3 (PMC10665425; doi:10.1038/s41419-023-06280-3)
Supplement: Supplementary file 6 — Supplementary figure legends [file 41419_2023_6280_MOESM6_ESM.docx]

**Supplementary Figure Legends**

**Fig. S1 Exosomes from PDPN-transfected CAFs regulate the motility and viability of OSCC cells.**

**A-B** The migration **(A)** and invasion **(B)** of OSCC cells treated with exosomes isolated from PDPN-transfected CAFs was measured by transwell assay. Bar, 200μm. Student’s t test for two-group comparison: **P* < 0.05; ***P* < 0.01.

**C-D** The effect of exosomes isolated from PDPN-transfected CAFs in the growth rate of both SCC15 **(C)** and WSU-HN6 **(D)** cells was examined using CCK-8 assay. Student’s t test for two-group comparison: **P* < 0.05; ***P* < 0.01.

**E** The migration of WSU-HN6 cells direct co-cultured with GW4869 treated PDPN-transfected CAFs was measured by wound healing assay. Bar, 200 μm. one-way ANOVA for multi-group comparisons: **P* < 0.05; ***P* < 0.01.

**F** The colony formation of WSU-HN6 cells indirect co-culture with GW4869 treated PDPN-transfected CAFs was measured by colony formation assay. one-way ANOVA for multi-group comparisons: ***P* < 0.01.

**Fig. S2 The chromosome position and protein coding capability of lncRNA FTX.**

**A** The chromosomal location of FTX.

**B** Full-length FTX was cloned into a pcDNA 3.1 vector with/without RFP gene, and these plasmids were subsequently transfected into 293T cells. pcDNA3.1 vector with RFP gene served as a positive control. Western blotting was used to detect the expression of RFP.

**C** Protein coding potential of FTX was analyzed by six online tools.

**Fig. S3 Kaplan-Meier plots of lncRNA FTX expression in 40 cases of OSCC patients.**

Overall survival rate was performed by log-rank test. log-rank test: *P* < 0.05.

**Fig. S4 The establishment of lncRNA FTX-transfected OSCC cells.**

**A** FTX-overexpressed SCC15 cell was established by the transfection of FTX over-expression lentiviral plasmids. The expression of FTX in the transfected OSCC cells were detected by qRT-PCR. Student’s t test for two-group comparison: ***P* < 0.01.

**B** FTX-silenced WSU-HN6 cell was established by the transfection of short hairpin RNA (shRNA) targeting FTX. The expression of FTX in the transfected OSCC cells were detected by qRT-PCR. Student’s t test for two-group comparison: ***P* < 0.01.

**Fig. S5 The establishment of FEN1-transfected OSCC cells.**

**A** FEN1-overexpressed SCC15 cell was established by the transfection of FEN1 over-expression lentiviral plasmids. The expression of FEN1 in the transfected SCC15 cells were detected by western blotting and qRT-PCR. Student’s t test for two-group comparison: ***P* < 0.01.

**B** FEN1-silenced WSU-HN6 cell was established by the transfection of short hairpin RNA (shRNA) targeting FEN1. The expression of FEN1 in the transfected OSCC cells were detected by western blotting and qRT-PCR. Student’s t test for two-group comparison: ***P* < 0.01.
