# Supplementary material for: PDPN+ CAFs facilitate the motility of OSCC cells by inhibiting ferroptosis via transferring exosomal lncRNA FTX
Source: Cell Death Dis. 2023 Nov 22;14(11):759. doi: 10.1038/s41419-023-06280-3 (PMC10665425; doi:10.1038/s41419-023-06280-3)
Supplement: Supplementary file 7 — Supplementary Table 1 [file 41419_2023_6280_MOESM7_ESM.docx]

**Supplementary Table 1. The sequences of the following primers were used:**

| **Gene** | |  | | **Primer Sequence** | |
| --- | --- | --- | --- | --- | --- |
| FTX | F | | 5'-GAGGATGAATTACGGTTGCC -3' | |  |
|  | R | | 5'-CCTGAGATACCTGTTCCTTTGT -3' | |  |
| α-SMA | F | | 5'-CTGTTCCAGCCATCCTTCATC-3' | |  |
|  | R | | 5'-CCGTGATCTCCTTCTGCATT-3' | |  |
| FAP | F | | 5'-GGAAGTGCCTGTTCCAGCAATG-3' | |  |
|  | R | | 5'-TGTCTGCCAGTCTTCCCTGAAG-3' | |  |
| PDGFRβ | F | | 5'-CCGGAACAAACACACCTTCT-3' | |  |
|  | R | | 5'-TATCCATGTAGCCACCGTCA-3' | |  |
| Caveolin-1 | F | | 5'-AATACTGGTTTTACCGCTTGCT-3' | |  |
|  | R | | 5'-CATGGTACAACTGCCCAGATG-3' | |  |
| U1 | F | | 5'-CAGGGGAGATACCATGATCACGAAG-3' | |  |
|  | R | | 5'-CGCAGTCCCCCACTACCACAAAT-3' | |  |
| U6 | F | | 5'-CTCGCTTCGGCAGCACA-3' | |  |
|  | R | | 5'-AACGCTTCACGAATTTGCGT-3' | |  |
| FEN1 | F | | 5'-CTGTGGACCTCATCCAGAAGCA-3' | |  |
|  | R | | 5'-CCAGCACCTCAGGTTCCAAGA-3' | |  |
| PDPN | F | | 5′-TGACTCCAGGAACCAGCGAAG-3′ | |  |
|  | R | | 5′-GCGAATGCCTGTTACACTGTTGA-3′ | |  |
| ACSL4 | F | | 5′-AACCCAGAAAACTTGGGCATT-3′ | |  |
|  | R | | 5′-GTCGGCCAGTAGAACCACT-3′ | |  |
| GAPDH | F | | 5'-GGAGCGAGATCCCTCCAAAAT-3' | |  |
|  | R | | 5'-GGCTGTTGTCATACTTCTCATGG-3' | |  |
