# Supplementary material for: PDPN+ CAFs facilitate the motility of OSCC cells by inhibiting ferroptosis via transferring exosomal lncRNA FTX
Source: Cell Death Dis. 2023 Nov 22;14(11):759. doi: 10.1038/s41419-023-06280-3 (PMC10665425; doi:10.1038/s41419-023-06280-3)

PDPN^{+} CAFs facilitate the motility of OSCC cells by inhibiting ferroptosis via transferring exosomal lncRNA FTX

Original Western Blot

Fig.2B

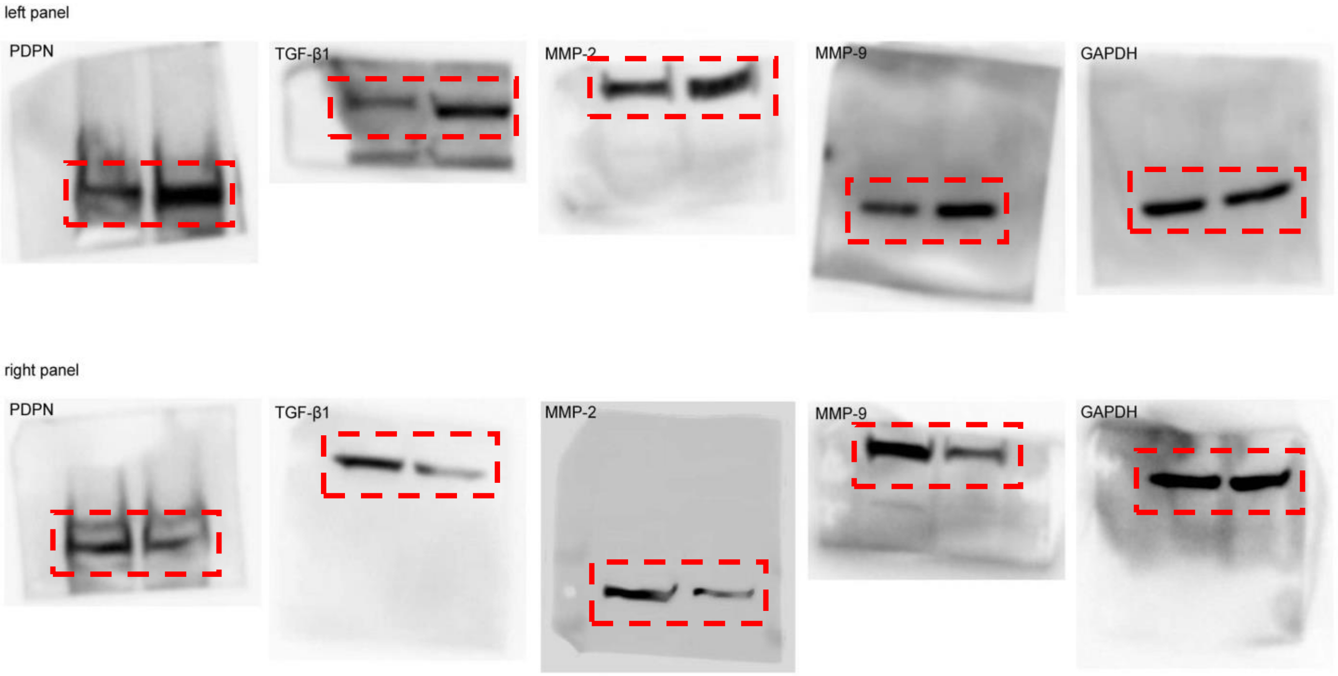
left panel
PDPN
TGF-β1
MMP-2
MMP-9
GAPDH
right panel
PDPN
TGF-β1
MMP-2
MMP-9
GAPDH

Fig.3C

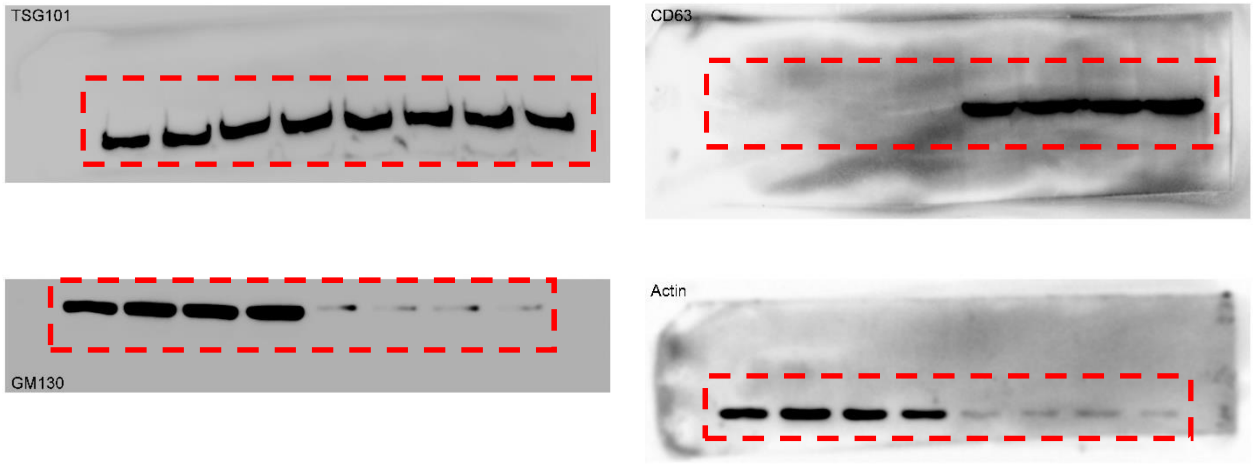
TSG101
CD63
GM130
Actin

Fig.4

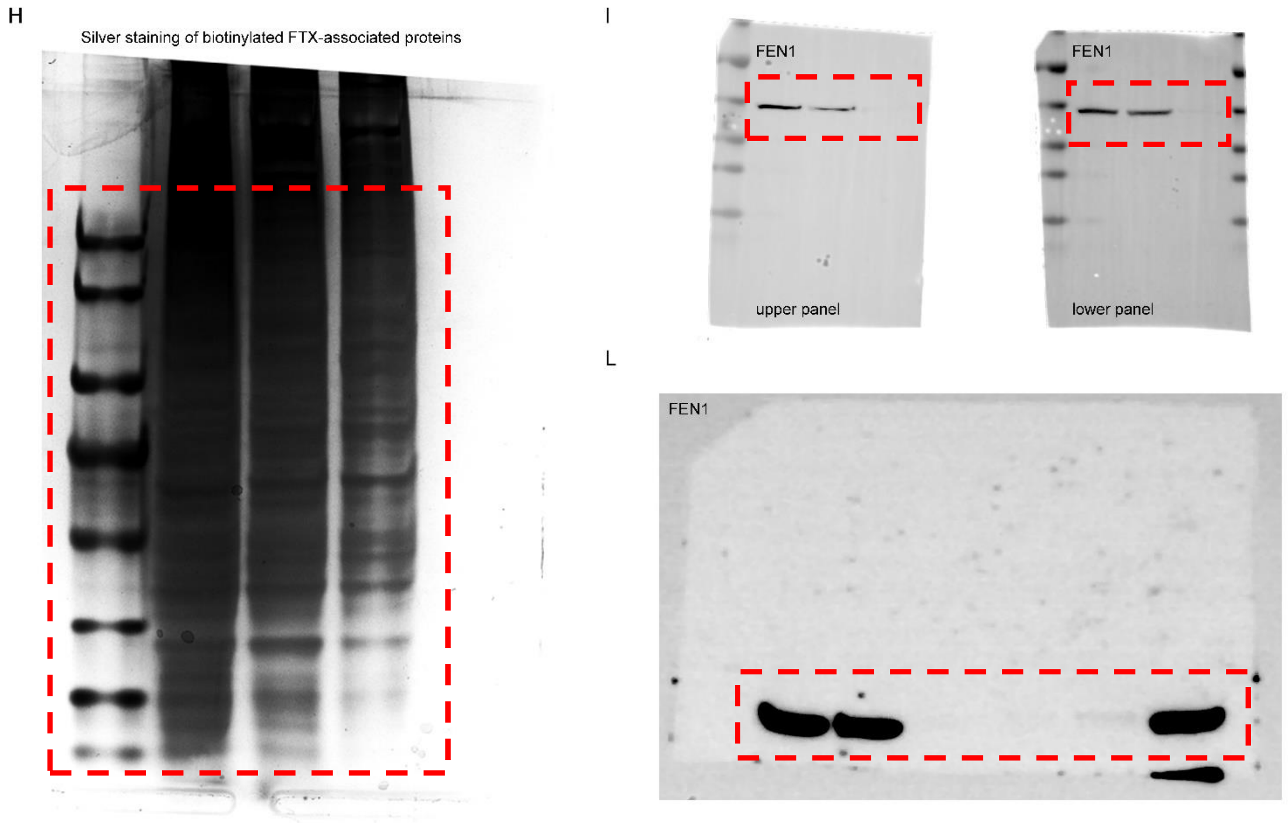
H
Silver staining of biotinylated FTX-associated proteins
I
FEN1
upper panel
FEN1
lower panel
L
FEN1

Fig.5

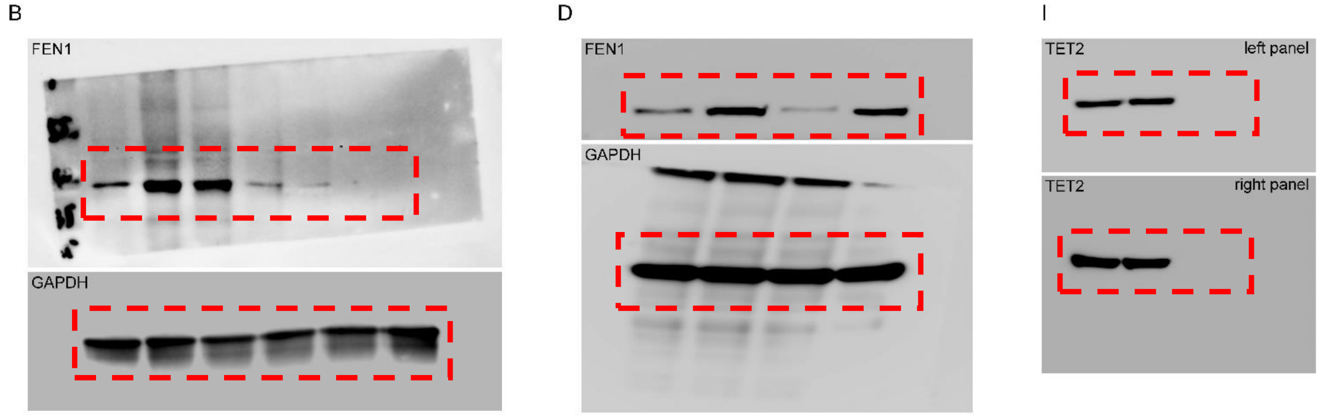
B
FEN1
GAPDH
D
FEN1
GAPDH
I
TET2
left panel
TET2
right panel

Fig.7

A

left panel

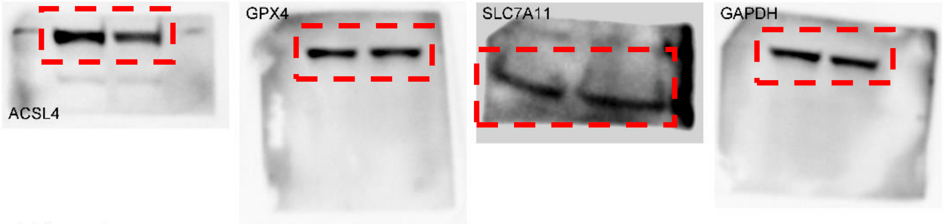

right panel

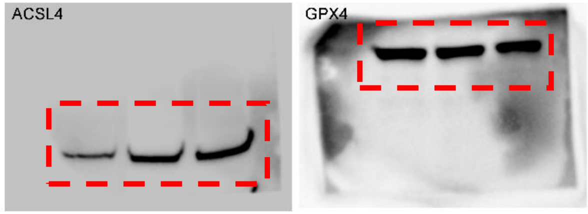

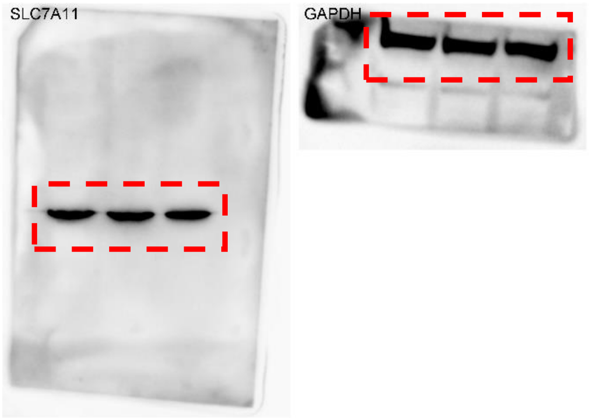

D

left panel

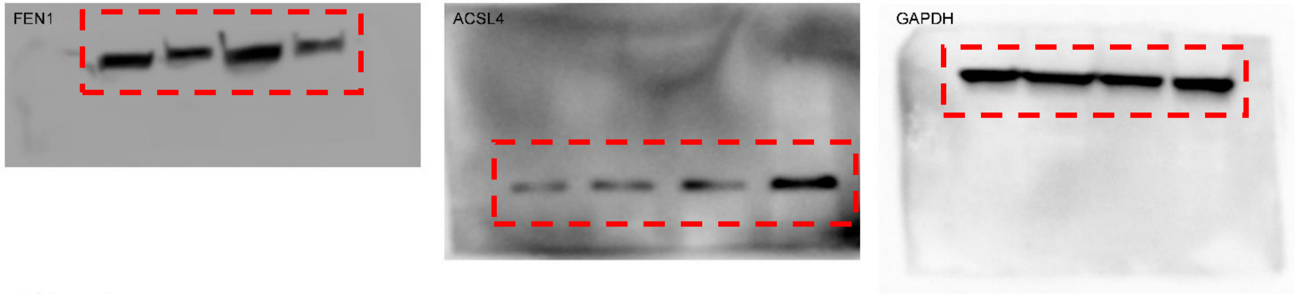

right panel

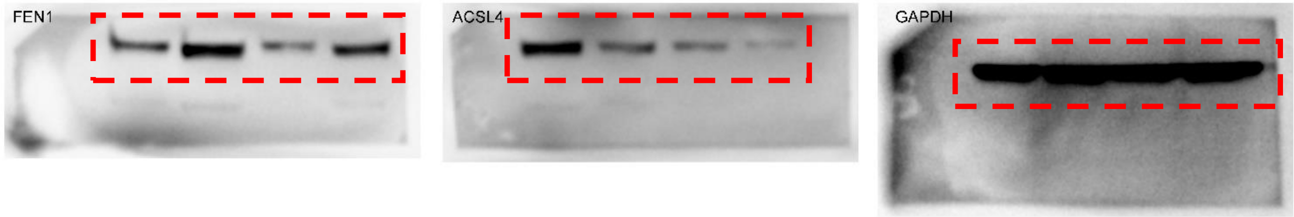

Fig.S2

B

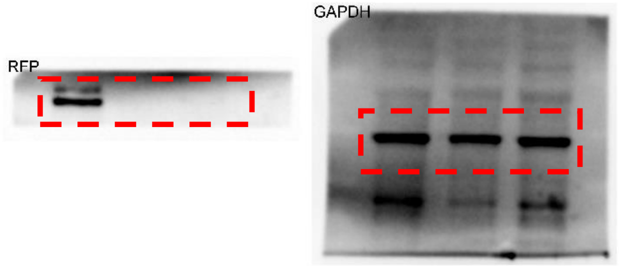

Fig.S5

A

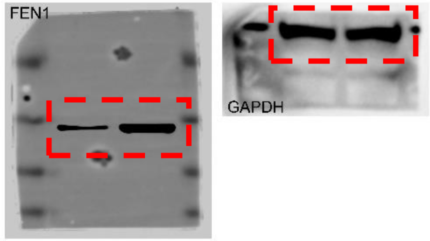

B

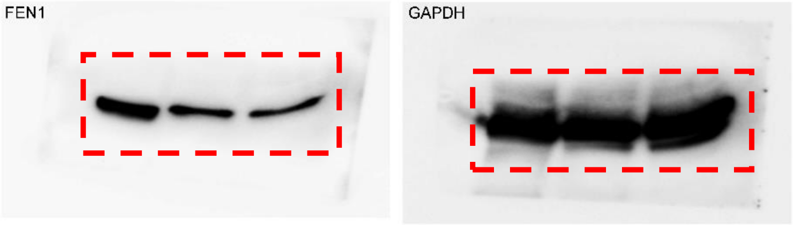

Supplement: Supplementary file 9 — Original western blotting Data File [file 41419_2023_6280_MOESM9_ESM.pdf]
